# Proton Reduction Catalyst-Grafted Covalent Organic Frameworks for Visible-Light-Driven Acceptorless Dehydrogenation of Cyclic Amines

**DOI:** 10.3390/ma19122602

**Published:** 2026-06-17

**Authors:** Bin Wang, Xinyu Shi, Qianping Wang, Xinrui Jiang, Wanqi Wang, Hui Chen

**Affiliations:** School of Chemistry and Chemical Engineering, Xi’an University of Architecture and Technology, Xi’an 710055, China; wb@xauat.edu.cn (B.W.); xyshi@xauat.edu.cn (X.S.); qpwang@xauat.edu.cn (Q.W.); xrjiang@xauat.edu.cn (X.J.); wqwang@xauat.edu.cn (W.W.)

**Keywords:** covalent organic frameworks, photocatalysis, proton-reduction catalyst acceptorless dehydrogenation, cyclic amines

## Abstract

The development of sustainable, noble-metal-free photocatalytic systems for acceptorless dehydrogenation (ADH) of cyclic amines remains a significant challenge. Herein, we report a novel heterogeneous photocatalyst constructed by covalently grafting a cobaloxime-based proton reduction catalyst onto a photosensitive covalent organic framework (PT-COF). The tailored PT-COF scaffold, featuring a donor–acceptor architecture and uncondensed amino groups, serves as both an efficient visible-light harvester and a porous support for cobalt active sites. The resulting Co-PT-COF hybrid exhibits excellent photocatalytic activity for the ADH of a wide range of cyclic amines, affording the corresponding N-heteroarenes in 62–95% yields under an Ar atmosphere at 28 °C with blue LED irradiation for 12 h in water. Notably, the catalyst demonstrates outstanding recyclability over five consecutive cycles with minimal loss of activity or cobalt leaching. Comprehensive photoelectrochemical and spectroscopic studies reveal that enhanced charge separation and efficient electron transfer from the photoexcited COF to the cobalt centers underpin the superior performance. Mechanistic investigations, including in situ EPR spectroscopy, confirm the involvement of *α*-amino radical intermediates in the catalytic cycle. This work establishes a sustainable platform for solar-driven dehydrogenation chemistry and provides a versatile blueprint for integrating molecular catalysts with photoactive frameworks.

## 1. Introduction

**Scheme 1:**
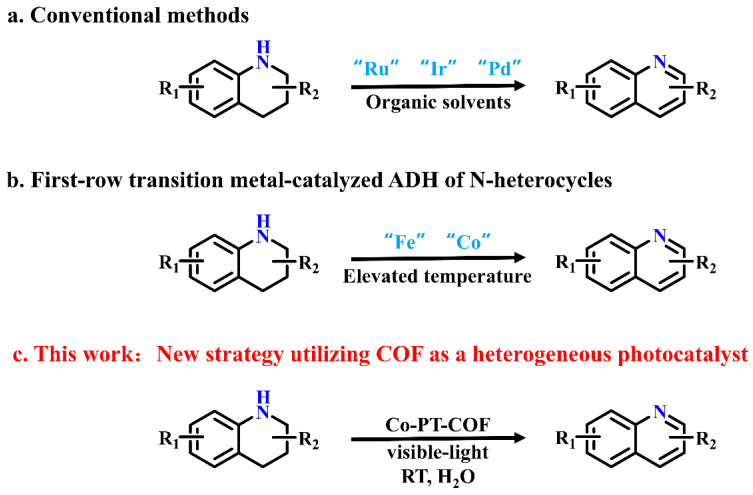
(**a**) conventional methods using Ru-, Ir-, or Pd-based catalysts in organic solvents. (**b**) first-row transition-metal-catalyzed acceptorless dehydrogenation of N-heterocycles under elevated temperature. (**c**) this work: visible-light-driven acceptorless dehydrogenation of N-heterocycles over Co-PT-COF in water at room temperature.

The pursuit of sustainable, atom-economical synthetic methodologies is a central theme in modern chemical research, driven by growing environmental concerns and the urgent need to transition to greener industrial processes [1]. Within this broad landscape, the catalytic dehydrogenation of nitrogen-containing heterocycles stands out as a transformation of both fundamental and practical significance [2,3]. Partially saturated N-heterocycles, such as tetrahydroquinolines, indolines, and piperidines, serve as pivotal precursors to their aromatic counterparts, N-heteroarenes, which are indispensable scaffolds in pharmaceuticals, agrochemicals, organic materials, and ligands [4,5,6]. Traditional access to these aromatic systems often relies on stoichiometric oxidants (such as 2,3-Dichloro-5,6-dicyano-1,4-benzoquinone) or harsh conditions, generating substantial waste [7,8,9]. In contrast, catalytic acceptorless dehydrogenation (ADH) offers an elegant and sustainable alternative [10,11]. This process involves the direct removal of two hydrogen atoms from the substrate, with molecular hydrogen (H_2_) as the sole byproduct [12]. Thus, ADH aligns perfectly with the principles of green chemistry by maximizing atom economy, avoiding external oxidants, and producing clean H_2_ fuel, thereby coupling fine chemical synthesis with renewable energy generation. Despite its conceptual appeal, the practical implementation of ADH poses significant thermodynamic and kinetic challenges [13,14,15,16]. The cleavage of two C-H or N-H bonds is inherently uphill, often requiring high temperatures (typically >150 °C) to proceed efficiently [17]. Pioneering work using homogeneous catalysts based on noble metals (e.g., Ru, Ir, Pd) has demonstrated feasibility, but these systems are hampered by high cost, potential metal toxicity, and frequent requirement for volatile organic solvents (VOCs) (Scheme 1a) [18,19,20]. More recently, catalysts based on earth-abundant first-row transition metals, particularly iron and cobalt, have emerged as promising alternatives for the thermal ADH of N-heterocycles (Scheme 1b) [21,22,23]. However, these systems still predominantly operate under energy-intensive thermal conditions, which can lead to undesired side reactions, catalyst decomposition, and limited functional group tolerance. Furthermore, the homogeneous nature of these catalysts complicates product separation and catalyst recycling, posing barriers to large-scale application.

Photocatalysis has emerged as a powerful strategy to overcome the kinetic barriers of thermal processes by harnessing light energy to drive chemical transformations under mild, often ambient conditions [24,25,26,27]. In the context of ADH, seminal work has utilized noble-metal photosensitizers like [Ru(bpy)_3_]^2+^ in conjunction with proton-reduction catalysts to achieve dehydrogenation with visible light [28]. While effective, this approach perpetuates the reliance on scarce and expensive ruthenium. Moreover, the homogeneous dual-catalyst systems can suffer from diffusion-limited electron transfer and face challenges in catalyst separation. Therefore, the development of integrated, heterogeneous photocatalytic systems that combine light-harvesting, substrate activation, and proton reduction within a single, recyclable material is a highly desirable yet underexplored goal.

More recently, porous framework materials, including metal–organic frameworks (MOFs) and covalent organic frameworks (COFs), have been explored for photocatalytic dehydrogenation-related reactions of N-heterocycles. For example, MOF-based photocatalysts have enabled visible-light-driven acceptorless dehydrogenation of tetrahydroquinoline and other N-heterocycles with concomitant H_2_ evolution [29,30]. In parallel, COF-based photocatalysts have also been applied to photocatalytic dehydrogenative cross-coupling and oxidative dehydrogenation reactions of N-heterocycles [31,32]. These studies demonstrate that porous framework materials can integrate visible-light absorption, substrate activation, and catalytic dehydrogenation functions within recyclable solid photocatalysts, providing useful guidance for the design of heterogeneous photocatalytic ADH systems. Also, Covalent organic frameworks (COFs) have recently ascended as a revolutionary class of porous, crystalline organic polymers with immense potential in photocatalysis [33]. Their designability allows for precise tuning of optical and electronic properties through the selection of *π*-conjugated building blocks, enabling strong visible-light absorption [34,35]. Their permanent, ordered nanoporosity provides a high surface area and well-defined channels that can facilitate substrate diffusion, concentration, and pre-organization, factors crucial for efficient catalysis [36]. Furthermore, their robust covalent linkage confers excellent chemical stability in various media, including water [37]. These attributes make COFs ideal platforms to serve as both photosensitizers and solid supports for anchoring molecular catalysts [38,39,40]. Additionally, cobalt catalysis has attracted increasing attention in photoredox catalysis and visible-light-driven organic synthesis. For example, Dutta and Breit reported a light-mediated photoredox/cobalt dual-catalytic system for the synthesis of multisubstituted enals [41], while Chi et al. summarized recent advances in photoredox-mediated cobalt catalysis for non-asymmetric and asymmetric synthesis [42], highlighting the important role of cobalt catalysis in visible-light-driven transformations.

Inspired by the promising developments in the use of COFs in photosynthesis, recent advances in cobalt-mediated photocatalysis, and our previous reports [43,44]. We hypothesized that COFs might form an ideal platform to combine photoredox and molecular catalysts to drive ADH reactions. Herein, cobaloximes were selected as the proton reduction molecular catalyst, which are well-established as highly efficient and robust molecular catalysts for the proton reduction reaction (HER), the key half-reaction in water splitting and many organic dehydrogenation processes [45,46]. In addition, the earth abundance, tunable redox properties, and proven catalytic activity of cobalt make cobalt complexes attractive candidates for integration with photoactive supports [47]. A [4 + 3] photosensitive COF (PT-COF) with free amino groups was chosen as both the photoredox catalyst and supporting scaffold. We engineer a heterogeneous bifunctional photocatalyst wherein the COF scaffold not only serves as a high-surface-area, visible-light-harvesting antenna but also as a rigid, porous matrix for the covalent immobilization of molecular catalyst. While He et al. demonstrated a homogeneous system combining a ruthenium photosensitizer with a cobalt catalyst for acceptorless dehydrogenation [20], and Banerjee et al. reported a cobaloxime–COF hybrid for sacrificial-donor-assisted H_2_ evolution [46], both systems suffer from limitations such as poor recyclability, use of noble metals, or reliance on sacrificial reagents. In contrast, our work uniquely integrates the acceptorless dehydrogenation (ADH) of cyclic amines with proton reduction into a single, heterogeneous, and recyclable framework by covalently grafting a cobaloxime catalyst onto a photoactive COF. This design eliminates the need for noble metals, volatile organic solvents, and sacrificial electron donors, while enabling efficient charge separation and catalyst recycling (Scheme 1c). Upon visible-light irradiation, the COF absorbs photons to generate electron–hole pairs. The photogenerated electrons are rapidly shuttled through the conjugated framework to the proximal cobaloxime sites. Concurrently, the photogenerated holes oxidize the amine substrate adsorbed within the COF pores, facilitating its concerted dehydrogenation to the corresponding N-heteroarene. In this synergetic system, the COF entirely replaces the need for expensive noble-metal complexes. The process operates efficiently in water under ambient temperature and neutral pH, eliminating VOCs and minimizing energy input. Significantly, covalent tethering of the cobalt catalyst within the COF pore ensures intimate contact with the photoexcited framework, minimizing charge recombination and enabling fast intramolecular electron transfer. Through detailed synthesis, characterization, and mechanistic studies, including spectroscopic investigations of charge carrier dynamics, we elucidate the synergistic interplay between the COF host and the grafted cobalt guest. This Co-PT-COF hybrid system demonstrates high activity and selectivity in the dehydrogenation of a range of cyclic amines, producing valuable N-heteroarenes in excellent yields. This study not only establishes a new, sustainable paradigm for solar-driven dehydrogenation chemistry but also provides a generalizable blueprint for the rational design of multifunctional porous photocatalysts by integrating molecular catalytic prowess with the modular architecture of COFs. The principles demonstrated here, modular component integration, nanoconfinement-enhanced catalysis, and the exploitation of light–matter interactions in porous solids, hold broad potential for application in other challenging bond-activation and energy-conversion reactions.

## 2. Materials and Methods

### 2.1. Chemicals

1,3,5-Trimethylbenzene and 1,4-dioxane were purchased from Aladdin Reagent Co., Ltd (Shanghai, China). 2,4,6-Tris(4-formylphenyl)-1,3,5-triazine, cobalt(II) chloride hexahydrate, dimethylglyoxime, 4-pyridinecarboxaldehyde, acetic acid, and triethylamine were obtained from Meryer Chemical Technology Co., Ltd (Shanghai, China). 4,4′,4″,4‴-(Pyrene-1,3,6,8-tetrayl)tetraaniline, 1,2,3,4-Tetrahydroquinoline, 1,2,3,4-Tetrahydroquinaldine, 3-Methyl-1,2,3,4-tetrahydroquinoline, 4-Methyl-1,2,3,4-tetrahydroquinoline, 6-Chloro-1,2,3,4-tetrahydroquinoline, 6-Bromo-1,2,3,4-tetrahydroquinoline, 6-Nitro-1,2,3,4-tetrahydroquinoline, Methyl 1,2,3,4-tetrahydroquinoline-6-carboxylate, 7-Amino-1,2,3,4-tetrahydroquinoline, 6-Methyl-1,2,3,4-tetrahydroquinoline, 2,3-Dimethyl-1,2,3,4-tetrahydroquinoxaline, Indoline, 2-Methylindoline, Ethyl indoline-2-carboxylate, and 5-Methoxyindoline were purchased from Anhui Zesheng Technology Co., Ltd (Anqing, China). All the chemicals were used as received without any further purification.

### 2.2. Synthesis of Cobaloxime-Based Proton Reduction Catalysts

Acetone (25 mL), dimethylglyoxime (10 mmol), and CoCl_2_·6H_2_O (5.2 mmol) were stirred in a 50 mL round-bottom flask at room temperature for 2 h under an oxygen atmosphere supplied by a balloon. After 2 h, the mixture was cooled at −15 °C for 12 h. The resulting precipitate was collected by filtration, rinsed with cold acetone, and air-dried to afford a green solid. We added triethylamine (2.5 mmol) to a suspension of the solid (2.5 mmol) in MeOH (50 mL) at room temperature, stirred the mixture for 1 h, then added 1 equivalent of 4-pyridinecarboxaldehyde and continued stirring for another 1 h. After 1 h, the mixture was cooled at −15 °C for 12 h. The precipitate was then collected by filtration, washed with cold methanol, and air-dried.

### 2.3. Synthesis of PT-COF

PT-COF was prepared using a conventional solvothermal method. Briefly, 4,4′,4″,4‴-(pyrene-1,3,6,8-tetrayl)tetraaniline (0.076 mmol, 43.2 mg), 2,4,6-tris(4-formylphenyl)-1,3,5-triazine (0.076 mmol, 30 mg), 1,3,5-trimethylbenzene (3 mL), and 1,4-dioxane (3 mL) were charged into a Pyrex glass tube. The mixture was subjected to ultrasonication for about 30 min to achieve homogeneous dispersion, followed by the addition of 0.6 mL of 6 M aqueous acetic acid solution under shaking conditions, and a further ultrasonication treatment for approximately 60 min. Subsequently, the reaction tube was rapidly frozen in a liquid nitrogen bath at 77 K, evacuated with an oil pump, and then hermetically sealed, with degassing performed via three freeze–pump–thaw cycles. After reaching room temperature, the mixture was heated at 120 °C for 72 h. We isolated the resulting precipitate by filtration through filter paper on a Buchner funnel and washed it thoroughly with tetrahydrofuran and anhydrous ethanol until the filtrate became colorless. Finally, vacuum drying of the solid at 70 °C afforded PT-COF.

### 2.4. Synthesis of Co-PT-COF

The prepared cobalt oxime (0.093 mmol, 40 mg) was dispersed in anhydrous ethanol (37.5 mL) in a round-bottom flask, and sonication was performed until a golden-yellow solution formed. PT-COF (0.022 mmol, 20 mg) was added and sonicated for 30 min, followed by addition of 6 M acetic acid (0.1 mL) and further sonication for 10 min. We refluxed the mixture with stirring at 80 °C for 12 h. The obtained solid was collected by filtration, washed with anhydrous ethanol (3 × 20 mL), and oven-dried at 70 °C overnight to yield Co-PT-COF.

### 2.5. General Procedure for Catalytic Acceptorless Dehydrogenation of N-Heterocycles

Under an Ar atmosphere, substrate (0.25 mmol), Co-PT-COF (10 mg), and degassed water (8.0 mL, Ar-purged for 30 min) were added to an oven-dried 20 mL headspace vial containing a magnetic stir bar, which was then sealed and placed on a magnetic stirrer. Then, the mixture was irradiated with blue LED strips positioned approximately 3 cm away, with constant stirring at room temperature. A cooling fan was used to keep the reaction temperature at 28 °C. After irradiation for 12 h, the mixture was filtered and subjected to GC-MS analysis. The GC-MS analysis was performed using an HP-5MS capillary column (30 m × 0.25 mm × 0.25 μm). The oven temperature program was as follows: an initial temperature of 50 °C was held for 2 min, then increased to 280 °C at a rate of 15 °C/min and held for 5 min. The injection volume was 1 μL, and toluene (0.1 mmol) was used as the internal standard.

## 3. Results and Discussion

**Scheme 2:**
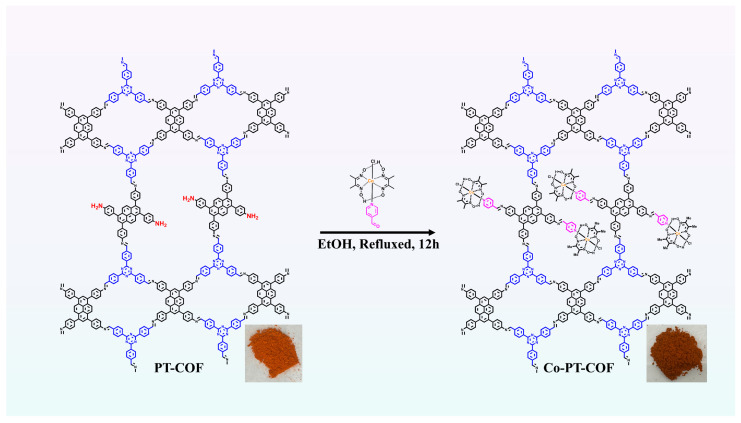
The synthesis process of Co-PT-COF.

The photosensitive COF scaffold (denoted as PT-COF) was initially synthesized under solvothermal conditions in a sealed ampule via a [4 + 3] Schiff-base condensation between 2,4,6-tris(4-formylphenyl)-1,3,5-triazine (TFP) and 4,4′,4″,4‴-(pyrene-1,3,6,8-tetrayl)tetraaniline (Py). The judicious selection of these planar building blocks promotes strong π-π stacking interactions between adjacent layers, thereby facilitating the formation of a highly crystalline framework [48]. Moreover, the integration of electron-deficient triazine units and electron-rich pyrene moieties engenders well-defined donor–acceptor (D-A) architectures within the COF backbone, establishing topologically ordered D-A heterojunctions. These heterojunctions provide spatially separated pathways for ambipolar charge transport, significantly enhancing photoconductivity and, consequently, the overall photocatalytic performance [49]. Importantly, the distinct connectivity of the tri- and tetratopic linkers in this [4 + 3] imine-linked COF, which adopts a *bex* net topology, results in the presence of uncondensed amino groups periodically arrayed within the pore channels. These accessible free amines serve as versatile anchoring sites for post-synthetic functionalization. Specifically, they enable the covalent immobilization of an aldehyde-functionalized cobalt-based proton reduction catalyst through a secondary Schiff-base condensation, yielding the composite material Co-PT-COF (Scheme 2). This covalent grafting strategy ensures the stable confinement of molecular cobalt catalysts within the COF nanopores while achieving a high and homogeneous dispersion throughout the framework. The structural integrity and robustness of PT-COF were assessed by exposing the material to various harsh chemical environments. Notably, the framework exhibits exceptional chemical stability, retaining its crystallinity without any detectable change in the powder X-ray diffraction (PXRD) pattern after immersion in common organic solvents, as well as in 1 M HCl and 1 M NaOH aqueous solutions for seven days (Figure S1). Following the covalent incorporation of the cobalt catalyst, the morphology of the resulting Co-PT-COF was investigated by scanning electron microscopy (SEM). As shown in Figure S2, both PT-COF and Co-PT-COF consist of aggregated nanorods assembled from textured nanosheets, a morphology characteristic of their analogous chemical architectures. Notably, the surface of the Co-PT-COF nanorods appears rougher than that of the pristine PT-COF, likely reflecting the successful anchoring of cobalt complexes onto the framework surface [50]. The crystal structure of Co-PT-COF was characterized by high-resolution transmission electron microscopy (HRTEM), the obtained image of which shows relatively clear lattice fringes (Figure S3a). Energy-dispersive X-ray (EDX) mapping reveals the homogeneous distribution of C, N, O, and Co elements, which provides preliminary evidence for the successful grafting of cobaloxime (Figure S3c–f). The cobalt loading was quantified by inductively coupled plasma mass spectrometry (ICP-MS), yielding a content of 1.69 ± 0.2 mmol g^−1^. This comprehensive set of characterizations confirms the successful construction of a robust, crystalline, and atomically dispersed cobalt-functionalized COF photocatalyst.

**Figure 1:**
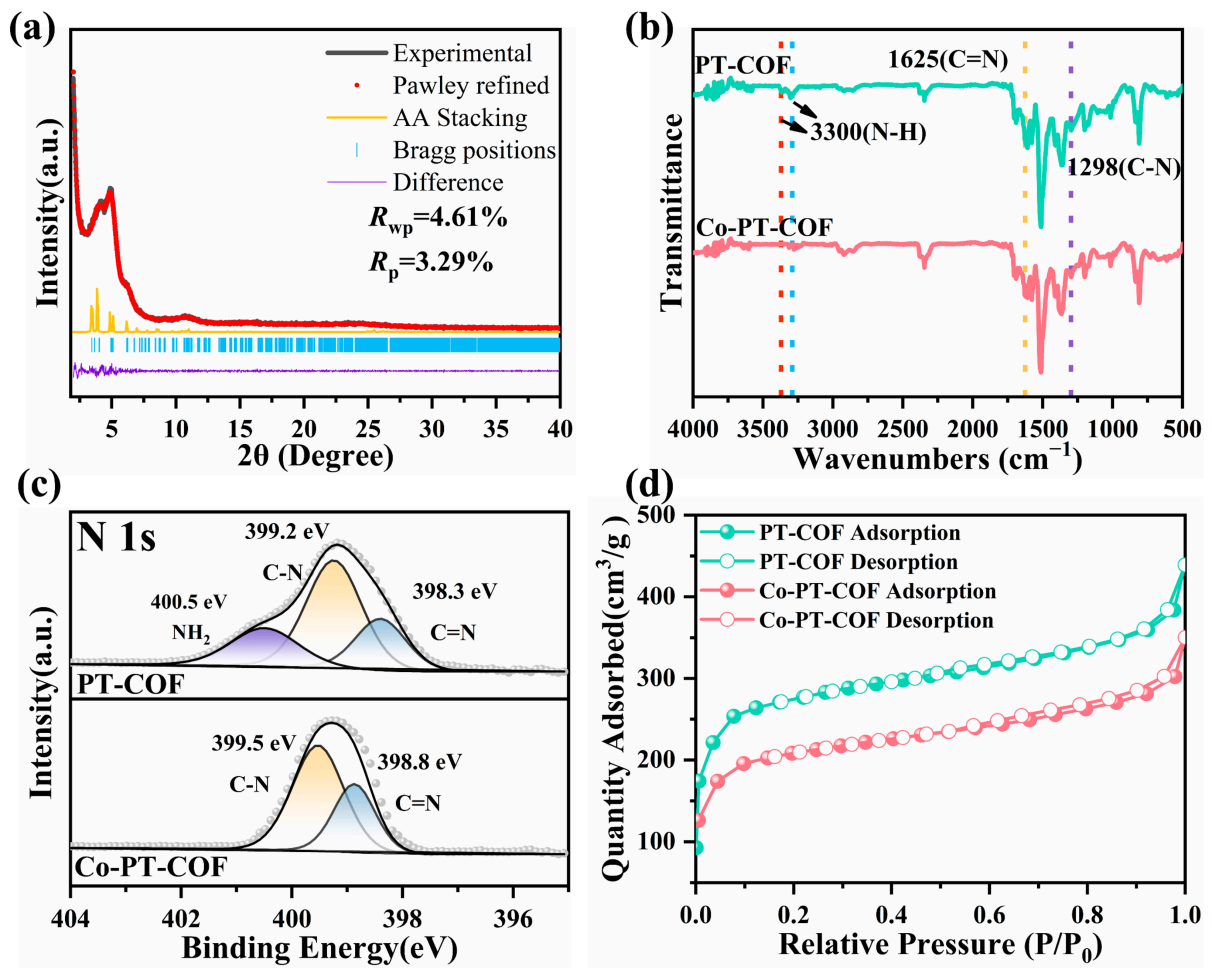
(**a**) Experimental and theoretical PXRD patterns of PT-COF. (**b**) FT-IR spectrum of PT-COF and Co-PT-COF. (**c**) N 1s XPS spectra of PT-COF and Co-PT-COF. (**d**) The N_2_ adsorption/desorption isotherms of PT-COF and Co-PT-COF at 77 K.

The crystalline structures of PT-COF and Co-PT-COF were elucidated using powder X-ray diffraction (PXRD) analysis (Figure 1a). The well-defined diffraction peaks attest to the high crystallinity of both materials. Characteristic reflections observed at 4.0°, 4.9°, 6.0°, and 10.7° correspond to the (110), (210), (310), and (130) crystallographic planes, respectively. The broadened peak centered at approximately 24° is attributed to π-π stacking interactions between adjacent COF layers, corresponding to the (001) plane. All diffraction features are consistent with the *P1* space group, indicative of a *bex*-type two-dimensional layered network. Structural simulations favor an eclipsed AA stacking configuration over a staggered AB arrangement. Pawley refinement of the experimental PXRD data yielded unit cell parameters of *a* = 53.272 Å, *b* = 25.047 Å, *c* = 3.838 Å, with *α* = 90.00°, *β* = 89.94°, and *γ* = 90.48°. The refined parameters show excellent agreement with the simulated pattern, as evidenced by the low weighted profile residual factor (*R_wp_* = 4.61%) and profile residual factor (*R_p_* = 3.29%). These low residual values indicate an excellent Pawley refinement fit and support the reliability of the proposed structural model. Notably, the PXRD pattern of Co-PT-COF closely resembles that of the parent PT-COF (Figure S4), confirming that the crystalline framework remains intact upon covalent grafting of the cobaloxime proton reduction catalyst.

The successful construction of PT-COF and its subsequent functionalization with the cobalt-based proton reduction catalyst were further corroborated by Fourier transform infrared (FT-IR) spectroscopy. As depicted in Figure 1b, both the pristine PT-COF and the post-synthetically modified Co-PT-COF exhibit a characteristic absorption band at 1625 cm^−1^, corresponding to the stretching vibration of the imine (C=N) linkage. This observation confirms the integrity of the COF backbone following the grafting procedure. Importantly, a distinctive doublet centered at approximately 3300 cm^−1^, attributable to the N-H stretching vibrations of primary amino groups (-NH_2_), is clearly visible in the spectrum of PT-COF. This feature arises from the uncondensed amine functionalities present within the COF pores. In the spectrum of Co-PT-COF, however, this doublet completely disappears, providing unambiguous spectroscopic evidence for the successful condensation of these free amines with the aldehyde-functionalized cobalt catalyst, thereby confirming its covalent anchoring onto the framework. Further insight into the chemical environment and bonding configuration was obtained through X-ray photoelectron spectroscopy (XPS, Figure S5). High-resolution N 1s core-level spectra of the pristine PT-COF were deconvoluted into three distinct components, corresponding to different nitrogen species (Figure 1c). The peaks centered at binding energies of approximately 398.3 eV, 399.2 eV, and 400.5 eV are assigned to imine nitrogen (C=N), aromatic amine nitrogen (C-N), and uncondensed primary amino groups (-NH_2_), respectively. Upon covalent immobilization of the cobalt catalyst, the spectral profile undergoes a significant change. Most notably, the component at ~400.5 eV, characteristic of the -NH_2_ groups, is completely absent in the N 1s spectrum of Co-PT-COF. This result is in excellent agreement with the FT-IR observations, collectively demonstrating the consumption of free amines through Schiff-base condensation with the aldehyde-functionalized cobalt complex. The combined spectroscopic evidence from FT-IR and XPS unequivocally confirms the successful grafting of the cobalt-based proton reduction catalyst onto the PT-COF scaffold, yielding the target Co-PT-COF hybrid material.

The permanent porosity of both the pristine PT-COF and the cobalt-functionalized Co-PT-COF was evaluated by measuring nitrogen adsorption–desorption isotherms at 77 K. As illustrated in Figure 1d, both materials exhibit a steep nitrogen uptake at low relative pressures (P/P_0_ < 0.1), a characteristic feature of microporous solids. This indicates that the microporous architecture of the COF scaffold is largely preserved following post-synthetic modification. Quantitatively, the Brunauer–Emmett–Teller (BET) surface area and the total pore volume (calculated at P/P_0_ = 0.97) decrease from 955.76 m^2^ g^−1^ and 0.645 cm^3^ g^−1^ for PT-COF to 726.74 m^2^ g^−1^ and 0.51 cm^3^ g^−1^ for Co-PT-COF, respectively. This reduction is anticipated and can be attributed to partial occupation of the COF nanopores by the covalently tethered cobalt-based proton-reduction catalyst, which occupies a portion of the void space within the channels. Pore-size distribution analysis, derived from nitrogen adsorption data using the quenched solid density functional theory (QSDFT) carbon model, reveals that both PT-COF and Co-PT-COF possess uniform micropores with diameters of approximately 1.97 nm (Figure S6). The retention of a well-defined pore size after catalyst grafting underscores the structural integrity of the framework and confirms that the covalent anchoring of cobalt complexes does not induce pore collapse or significant structural deformation. Importantly, despite the reduction in surface area and pore volume, the Co-PT-COF hybrid material maintains a permanent and accessible porous structure. This preserved porosity is critical for heterogeneous catalysis, as it ensures unhindered diffusion of substrate molecules to the active cobalt sites embedded within the COF channels, thereby facilitating efficient catalytic turnover [51]. In addition to permanent porosity, thermal stability is a paramount requirement for the practical application of any heterogeneous catalyst. The thermal behavior of both PT-COF and Co-PT-COF was investigated by thermogravimetric analysis (TGA) under a nitrogen atmosphere (Figure S7). The TGA profiles demonstrate that both materials exhibit exceptional thermal robustness, with no significant weight loss observed up to 450 °C. This high thermal stability ensures that the catalyst can withstand the conditions typically encountered during catalytic reactions, activation procedures, and potential regeneration steps, further affirming its suitability for sustained heterogeneous catalysis.

**Figure 2:**
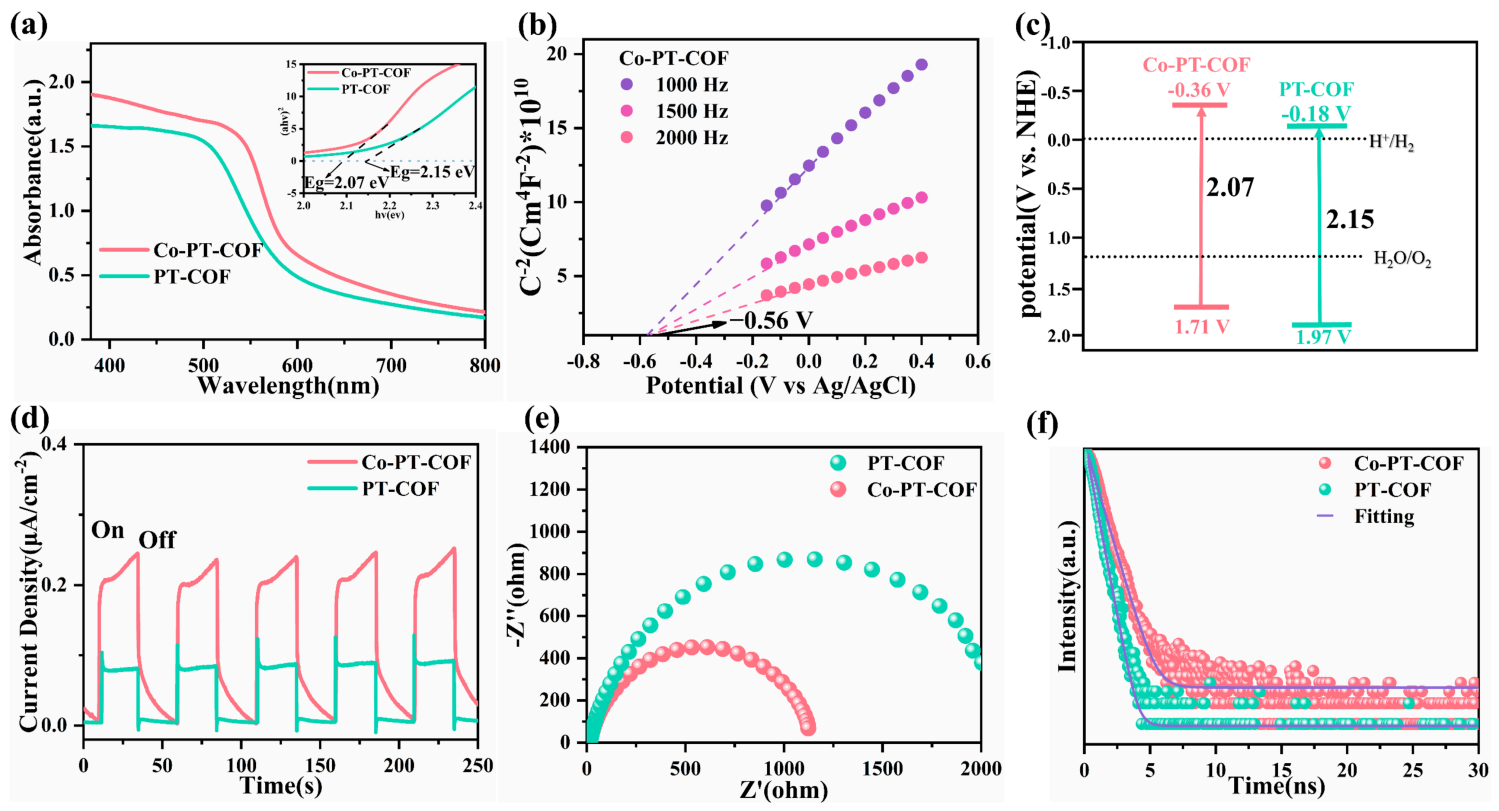
(**a**) Solid-state UV-Vis spectroscopy and (inset) Tauc-plot analysis of PT-COF and Co-PT-COF. (**b**) Mott–Schottky curves of Co-PT-COF. (**c**) Energy band alignment diagram of Co-PT-COF and PT-COF. (**d**) Transient photocurrent response of PT-COF and Co-PT-COF. (**e**) Electrochemical impedance spectroscopy (EIS) Nyquist plots of PT-COF and Co-PT-COF. (**f**) Time-resolved PL decay kinetics of PT-COF and Co-PT-COF at R.T.

The optical and electronic properties of PT-COF and its cobalt-functionalized analogue Co-PT-COF were systematically investigated to assess their suitability as visible-light-driven photocatalysts. Ultraviolet–visible diffuse reflectance spectroscopy (UV-Vis DRS) was employed to evaluate the light-harvesting capabilities of both materials. As depicted in Figure 2a, the absorption profiles of PT-COF and Co-PT-COF extend well beyond 500 nm into the visible region, indicating their capacity to harness a broad spectrum of solar irradiation. The corresponding optical band gaps (*E_g_*) were derived from Tauc plots, affording values of 2.15 eV for PT-COF and 2.07 eV for Co-PT-COF. Notably, these values are comparable to, or even narrower than, those of previously reported photocatalytic COFs such as LZU-190 (2.02 eV) [52], Py-Bd COF (2.21 eV) [53], and DS-COF (2.07 eV) [54], underscoring the competitive visible-light absorption of the present materials. The slightly reduced band gap observed for Co-PT-COF likely stems from electronic perturbations introduced upon cobalt grafting [55]. To elucidate the electronic band structure, Mott–Schottky (M-S) analysis was conducted at an AC frequency of 1 kHz, 1.5 kHz, and 2 kHz (Figure 2b and Figure S8). The positive slopes of the M-S plots confirm *n*-type semiconductor behavior for both PT-COF and Co-PT-COF. The flat-band potentials (*E_FB_*), determined from the x-intercepts of the linear regions, were estimated to be −0.38 V and −0.56 V vs. Ag/AgCl for PT-COF and Co-PT-COF, respectively. For *n*-type semiconductors, the conduction band (CB) edge is typically located approximately 0.1–0.2 V above the flat-band potential. Thus, the CB positions are approximated to be −0.18 V and −0.36 V vs. NHE. Using the optical band gaps obtained from Tauc plots, the valence band (VB) edges were calculated to be 1.97 V vs. NHE for PT-COF and 1.71 V vs. NHE for Co-PT-COF (Figure 2c). The anodic shift in the VB of Co-PT-COF relative to PT-COF reflects a modulation of the electronic structure upon catalyst immobilization.

A comprehensive suite of photoelectrochemical and spectroscopic techniques was employed to probe the charge carrier dynamics underpinning the enhanced photocatalytic potential of Co-PT-COF. Transient photocurrent response measurements (Figure 2d) revealed that Co-PT-COF generates a markedly higher photocurrent density than PT-COF under intermittent visible-light irradiation. This enhancement signifies more efficient photoinduced charge generation and separation within the hybrid material, minimizing energy-wasting recombination pathways. Electrochemical impedance spectroscopy (EIS) provided complementary insights. The Nyquist plot for Co-PT-COF (Figure 2e) exhibits a substantially reduced arc radius compared to that of PT-COF, indicating a lower charge transfer resistance at the electrode–electrolyte interface and, consequently, more facile interfacial charge migration. Steady-state photoluminescence (PL) spectroscopy further corroborated these findings (Figure S9). Upon excitation, Co-PT-COF displays pronounced fluorescence quenching relative to pristine PT-COF, directly evidencing the suppression of radiative electron–hole recombination. This quenching is attributed to the introduction of non-radiative decay channels facilitated by electron transfer from the photoexcited COF scaffold to the grafted cobalt centers. Time-resolved PL (TRPL) decay kinetics provided a quantitative assessment of this phenomenon (Figure 2f). The fluorescence decays, monitored at 410 nm, were best fitted with a biexponential function (R^2^ > 0.99), revealing a significantly extended average excited-state lifetime (*τ_avg_*) for Co-PT-COF (3.21 ns) compared to PT-COF (1.98 ns). This prolonged lifetime is diagnostic of efficient interfacial charge separation. The decelerated decay kinetics arise from the rapid extraction of photogenerated electrons by the cobalt clusters, which effectively competes with direct band-edge recombination. Collectively, these photoelectrochemical and spectroscopic analyses unequivocally demonstrate that the covalent integration of cobalt-based proton-reduction catalysts into the PT-COF framework enhances charge-separation dynamics, a critical prerequisite for high-performance photocatalysis.

Our results demonstrate that the Co-PT-COF possesses a high crystallinity, permanent porosity, outstanding stability, and promising photoredox properties. To validate its photocatalytic properties, a visible-light-driven ADH reaction was carried out. First, 2-Methyl-1,2,3,4-tetrahydroquinoline (1a) was used as a model substrate for the optimization of the reaction conditions (Table 1). Under the standard conditions employing 1a (0.25 mmol), Co-PT-COF (10 mg), water (8 mL) under Ar atmosphere, and visible-light irradiation from a 34 W blue LED at room temperature for 12 h, an excellent GC yield of 95% was achieved (entry 1). Control experiments confirmed the essential role of both light and the integrated catalyst. Reactions performed in the dark (entry 2), in the absence of Co-PT-COF (entry 3), or with its non-cobalt analogue PT-COF (entry 4) resulted in no reaction. Similarly, using only the molecular cobalt complex cobaloxime (entry 5) also gave no product, indicating that the photosensitive COF scaffold is crucial for catalytic activity. Notably, a physical mixture of Cobaloxime and PT-COF afforded a moderate yield of ~52% (entry 6), suggesting that proximity and integration of the cobalt sites within the COF structure enhance catalytic performance. In contrast, a mixture of Co(NO_3_)_2_·6H_2_O and PT-COF was ineffective (entry 7), underscoring the importance of the structured cobalt-oxime environment. In addition, no desired product was detected upon adding TEMPO (1 equiv.) (entry 8), suggesting the involvement of radical intermediates. Solvent screening revealed that water is optimal for this transformation (Table S2). While methanol and ethanol gave good yields (~86% and ~92%, respectively; Table S2, entries 1,2), all other tested organic solvents, including acetone, acetonitrile, toluene, THF, 1,4-dioxane, DMF, DMSO, and NMP, resulted in no reaction (Table S2, entries 3–10). This strong solvent dependence highlights the unique compatibility of the Co-PT-COF catalytic system with aqueous media, aligning with green chemistry principles. In summary, the optimal protocol employs Co-PT-COF as a heterogeneous photocatalyst, water as solvent, and visible-light irradiation under argon atmosphere, providing high-yielding dehydrogenation under mild and environmentally benign conditions.

With the optimized reaction conditions established, the substrate scope was systematically expanded to evaluate the broad applicability of the Co-PT-COF catalyst in ADH reactions. A diverse array of tetrahydroquinoline derivatives bearing either electron-donating or electron-withdrawing substituents, including methyl, methoxy, halogen, carbomethoxy, and nitro groups, was investigated. As summarized in Table 2, a range of structurally varied tetrahydroquinolines underwent smooth dehydrogenation to afford the corresponding N-heteroarenes in moderate to excellent yields, ranging from 62% to 95%. Comparative analysis of substrates 2a, 2b, and 2c revealed that the positional isomerism of the substituent exerts no significant influence on the reaction outcome, indicating good steric tolerance of the catalytic system. Notably, substrates equipped with electron-donating groups exhibited enhanced reactivity, furnishing the desired dehydrogenated products in superior yields relative to their electron-deficient counterparts. For instance, the unsubstituted tetrahydroquinoline delivered product 2d in 83% yield, while derivatives featuring electron-donating amino or methoxy groups (2f and 2g) were converted with exceptional efficiency, affording yields of 86% and 92%, respectively. In contrast, substrates bearing electron-withdrawing functionalities such as bromo, chloro, carbomethoxy, and nitro groups (2h–2k) participated readily in the ADH process under standard conditions, albeit providing the corresponding products in moderate yields ranging from 66% to 78%. Encouraged by these results, the synthetic utility of the Co-PT-COF catalyst was further extended to other important classes of N-heterocyclic compounds, including quinoxalines, and indoles, privileged scaffolds prevalent in pharmaceuticals, natural products, and biologically active molecules. Gratifyingly, substituted tetrahydroquinoxalines underwent efficient dehydrogenation under the optimized conditions, affording 2,3-dimethylquinoxaline (2l) in 82% yield. Similarly, indoline derivatives proved to be competent substrates: unsubstituted indoline, 2-methylindoline, 5-methoxyindoline, and methyl indoline-2-carboxylate were smoothly converted to their corresponding aromatic products 2m, 2n, 2o, and 2p in 88%, 90%, 81%, and 62% yields, respectively. These results underscore the versatility and robustness of the Co-PT-COF catalyst in promoting ADH reactions across a broad spectrum of nitrogen-containing heterocycles. The identities of the dehydrogenated products were confirmed by GC–MS comparison with authentic standards, and all products were further characterized by ^1^H NMR spectroscopy (see Supplementary Material).

**Figure 3:**
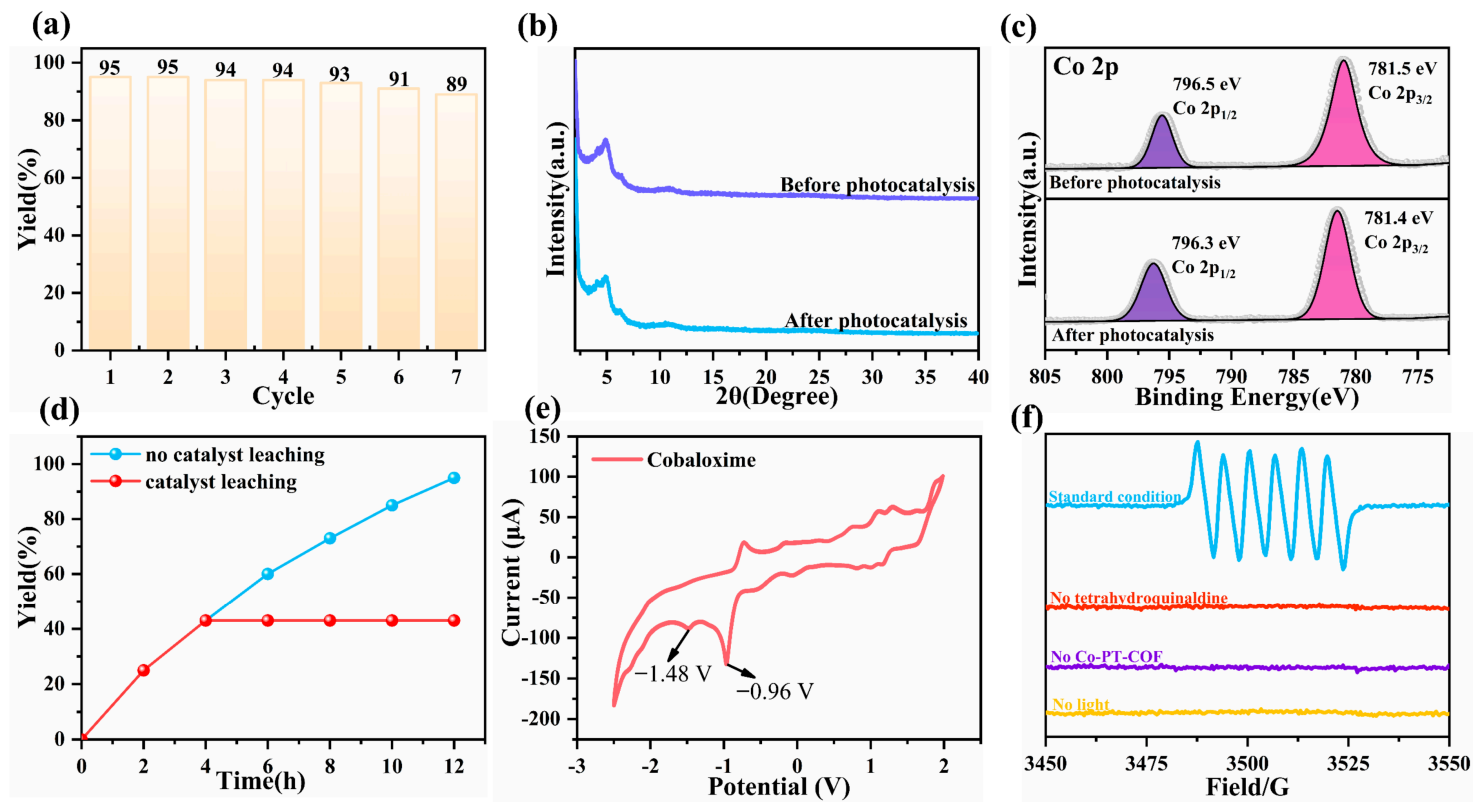
(**a**) Yield variation of Co-PT-COF in cyclic catalysis experiments. (**b**) PXRD pattern of Co-PT-COF before and after photocatalysis. (**c**) Co 2p XPS spectra of Co-PT-COF before and after photocatalysis. (**d**) blank without solid catalyst. (**e**) Cyclic voltammogram of Cobaloxime measured in MeCN. (**f**) EPR spectra recorded under various conditions.

The recyclability of heterogeneous catalysts constitutes a critical parameter for their practical implementation. Accordingly, the model reaction (Table 1, entry 1) was employed to evaluate the reusability of the Co-PT-COF catalyst under the optimized reaction conditions. As illustrated in Figure 3a, the catalyst could be readily recovered and reused for at least seven consecutive cycles without any discernible loss in catalytic activity, underscoring its excellent operational stability. Inductively coupled plasma mass spectrometry (ICP-MS) analysis of the reaction mixture after five cycles revealed no detectable cobalt leaching, confirming the robust covalent anchoring of the active sites within the COF framework. Further evidence for the structural integrity of the recycled catalyst was provided by PXRD, XPS, and FT-IR spectroscopy. The PXRD pattern of Co-PT-COF after multiple catalytic runs remained virtually unchanged from that of the fresh catalyst, indicating that the crystalline framework was largely preserved throughout the recycling process (Figure 3b). The Co 2p XPS spectrum of the recovered Co-PT-COF showed negligible changes compared with that of the fresh sample. The Co 2p_3/2_ and Co 2p_1/2_ peaks remained at nearly identical binding energies before and after photocatalysis, indicating that the chemical state and coordination environment of the Co sites were largely preserved during the reaction (Figure 3c). Similarly, the FT-IR spectrum of the recovered material retained all characteristic absorption bands, including the imine (C=N) stretch at 1625 cm^−1^, with no evidence of structural degradation (Figure S11). These observations collectively demonstrate that the covalent grafting strategy confers exceptional robustness, safeguarding both the crystallinity and the chemical functionality of the catalyst under prolonged reaction conditions. To unequivocally establish the heterogeneous nature of the catalysis, a leaching test was performed (Figure 3d). The reaction was halted after 4 h, at which point the solid catalyst was removed by hot filtration, and the filtrate was allowed to continue stirring under standard reaction conditions. No further conversion of the substrate was observed in the absence of the solid catalyst, confirming that the active cobalt species remain firmly immobilized on the COF support and that no catalytically competent species leach into the solution phase. Collectively, these results demonstrate that Co-PT-COF not only exhibits high photocatalytic performance but also outstanding recyclability and structural stability, positioning it as a promising, sustainable heterogeneous photocatalyst for organic transformations.

To elucidate the underlying reaction mechanism governing the ADH over Co-PT-COF, a combination of photophysical, electrochemical, and spectroscopic investigations was undertaken. Based on the energy band structure analysis detailed earlier, the *E_VB_* and *E_CB_* of Co-PT-COF were determined to be +1.71 V and −0.36 V vs. NHE, respectively. Cyclic voltammetry (CV) measurements revealed that the oxidation potential of the model substrate, tetrahydroquinaldine, lies at approximately +0.81 V vs. NHE, a value considerably lower than the VB potential of the COF. This energetic alignment suggests that direct hole transfer from the photoexcited COF to the substrate is thermodynamically feasible. Furthermore, the reduction potential of the cobalt-based proton reduction catalyst (*E*^III/II^) was measured to be −0.96 V vs. NHE (Figure 3e), positioning it appropriately for accepting electrons from the conduction band of the photoexcited COF. Steady-state emission quenching experiments further supported the electron-transfer process between PT-COF and the cobalt catalytic centers. With increasing cobaloxime concentration, the fluorescence intensity of PT-COF gradually decreased, indicating that cobaloxime could efficiently quench the excited state of PT-COF. Stern–Volmer analysis showed a good linear relationship between *I*_0_/*I* and the cobaloxime concentration, giving a *K*_SV_ value of 7.63 × 10^3^ M^−1^ with an R^2^ value of 0.99024 (Figure S12). Taken together, the steady-state emission quenching results and the favorable redox-potential alignment provide strong support for the concerted electron-transfer pathway in this system. Upon photoexcitation, the PT-COF scaffold can transfer an electron to the Co^III^ center, reducing it to Co^II^, while the resulting hole on the COF is simultaneously filled by an electron from the tetrahydroquinoline substrate, thereby initiating substrate oxidation.

Unambiguous evidence for the generation of key radical intermediates was obtained through in situ electron paramagnetic resonance (EPR) spectroscopy. Upon irradiation of a mixture containing tetrahydroquinaldine, Co-PT-COF, and the spin-trapping agent 5,5-dimethyl-1-pyrroline-N-oxide (DMPO) in aqueous medium under irradiation by a blue LED for 120 s, a well-resolved EPR signal characteristic of a DMPO-trapped *α*-amino radical adduct was observed (Figure 3f). The hyperfine coupling constants (α_N1_ = 14.50 G, α_H_ = 20.54 G, *g* = 2.006) are in excellent agreement with literature values for such species [56], confirming the formation of an *α*-amino radical intermediate derived from single-electron oxidation of the substrate. Additionally, the evolution of molecular hydrogen (H_2_) throughout the reaction was confirmed by gas chromatography (GC) analysis (Figure S13), corroborating the proton reduction function of the cobalt catalyst.

**Figure 4:**
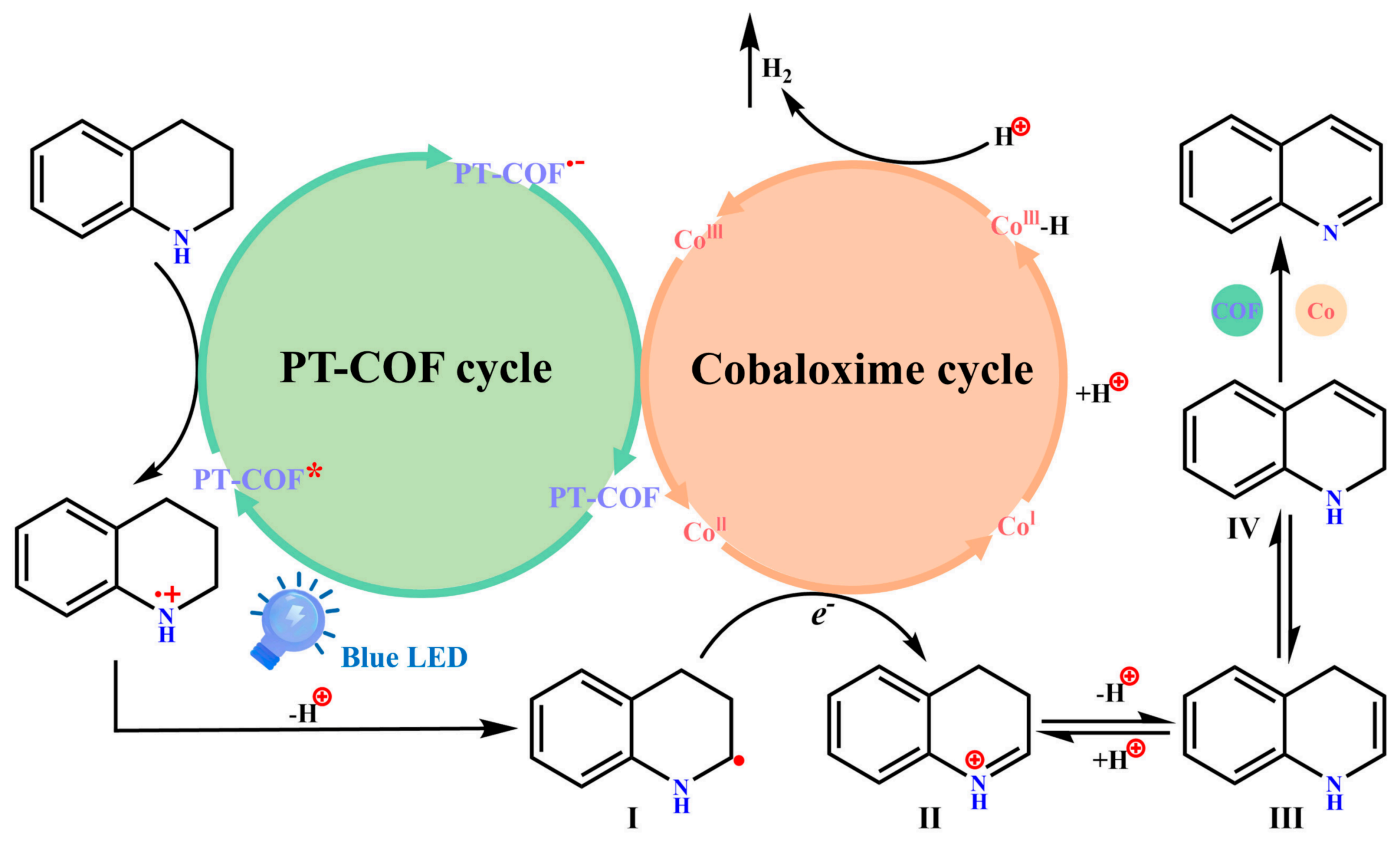
Proposed mechanism of Co-PT-COF-catalyzed acceptorless dehydrogenation of cyclic amines reaction.

Integrating these experimental findings with previously established mechanistic paradigms [57], a plausible catalytic cycle for the ADH reaction is proposed (Figure 4). The cycle is characterized by an intricate interplay between the photoactive COF scaffold and the cobalt catalytic centers, mediated by sequential electron and radical transfers. Upon visible-light irradiation, the photosensitive PT-COF is promoted to its excited state (PT-COF*). This excited species then engages in a single-electron transfer (SET) event with the tetrahydroquinoline substrate, effecting its oxidation to generate an *α*-amino radical intermediate (I) and a reduced COF species (PT-COF^•−^). The latter subsequently transfers an electron to the Co^III^ center, reducing it to Co^II^ while simultaneously regenerating the ground-state PT-COF to complete its photocatalytic cycle. In parallel, the *α*-amino radical (I) undergoes further oxidation by the Co^II^ species, yielding an iminium ion intermediate (II) and concomitantly reducing Co^II^ to Co^I^. This highly reduced cobalt center then reacts with a proton derived from the aqueous medium to form a Co^III^-hydride species ([Co^III^-H]). Protonation of this hydride by a second proton liberates molecular hydrogen (H_2_) and regenerates the active cobalt catalyst, closing the proton reduction cycle. Concurrently, the iminium intermediate (II) undergoes deprotonation and isomerization to afford the corresponding enamine (III) and subsequently 1,2-dihydroquinoline (IV). This partially unsaturated intermediate is then subjected to iterative oxidation and cobalt-mediated desaturation steps, presumably involving further SET events and hydride transfer processes, ultimately furnishing the fully aromatized quinoline product.

## 4. Conclusions

In summary, we have successfully developed a novel heterogeneous photocatalyst by covalently grafting a cobaloxime-based proton reduction catalyst onto a photosensitive PT-COF. The judiciously designed PT-COF scaffold, constructed from electron-deficient triazine and electron-rich pyrene building blocks, exhibits a highly crystalline structure with permanent porosity, exceptional chemical stability, and favorable optical properties. The strategic covalent immobilization of cobalt active sites within the COF nanopores ensures atomic-level dispersion, prevents metal leaching, and establishes intimate electronic communication between the photoactive framework and the catalytic centers. The resulting Co-PT-COF hybrid material demonstrates outstanding photocatalytic performance in the acceptorless dehydrogenation of a wide range of cyclic amines, including tetrahydroquinolines, indolines, and quinoxalines, affording the corresponding N-heteroarenes in moderate to excellent yields (62–95%) under mild, aqueous, and visible-light-driven conditions. The catalyst exhibits remarkable recyclability, retaining its crystallinity, structural integrity, and catalytic activity over at least five consecutive cycles without detectable cobalt leaching, underscoring its robustness and heterogeneous nature. Comprehensive photophysical and electrochemical investigations revealed that the enhanced photocatalytic activity originates from efficient charge separation and transfer dynamics. The PT-COF framework functions as an effective light-harvesting antenna, while the grafted cobalt centers serve as both electron sinks and proton reduction sites. Mechanistic studies, including radical scavenging experiments and in situ EPR spectroscopy, elucidated a synergistic catalytic cycle involving *α*-amino radical intermediates, with the cobalt sites facilitating proton reduction to molecular hydrogen. This work establishes a sustainable and noble-metal-free platform for visible-light-driven dehydrogenation chemistry, offering a compelling alternative to conventional thermal and homogeneous catalytic systems. The modular design strategy, integrating molecular proton reduction catalysts with photoactive porous frameworks, provides a versatile blueprint for developing advanced multifunctional materials for challenging organic transformations and clean energy conversion applications.

## Figures and Tables

**Table 1:** Optimization of the reaction conditions.

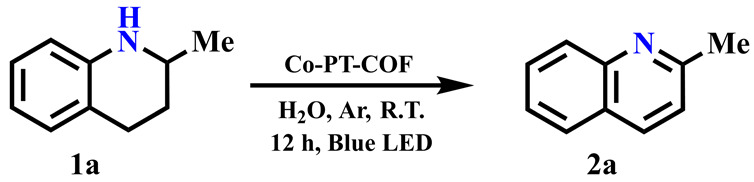		
Entry	Variation from the Standard Conditions	GC Yield ^[b]^
1	Standard conditions ^[a]^	~95%
2	No light (dark)	N. R.
3	No Co-PT-COF	N. R.
4	PT-COF instead of Co-PT-COF	N. R.
5	Cobaloxime instead of Co-PT-COF	N. R.
6	The physical mixture of Cobaloxime and PT-COF	~52%
7	The physical mixture of Co(NO_3_)_2_·6H_2_O and PT-COF	N. R.
8	Additional addition of TEMPO	N. R.

^[a]^ Reaction conditions: 1a (0.25 mmol), Co-PT-COF (10 mg), water (8 mL), Ar atmosphere, visible-light irradiation from blue LED at room temperature for 12 h. For entry 8, TEMPO (1 equiv.) was added under otherwise identical conditions. ^[b]^ GC yield using toluene as an internal standard. N.R. = No Reaction.

**Table 2:** Co-PT-COF photocatalytic dehydrogenation of cyclic amines.

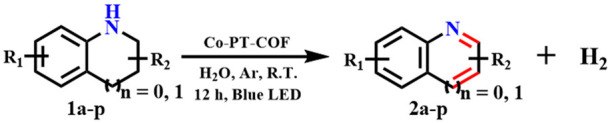
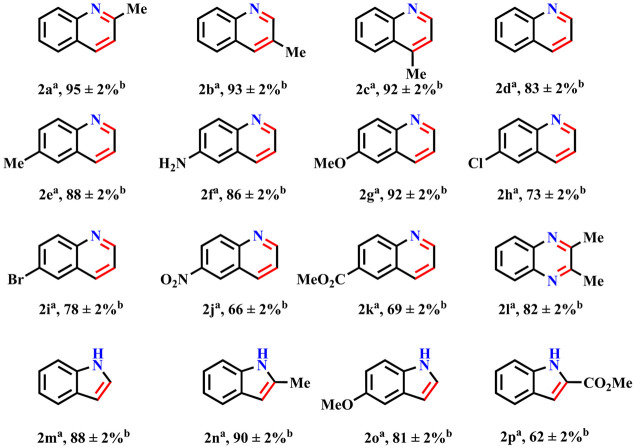

^a^ Reaction conditions: 1a-p (0.25 mmol), Co-PT-COF (10 mg), water (8 mL), Ar atmosphere, visible-light irradiation from blue LED at room temperature for 12 h. ^b^ GC-MS yield using toluene as an internal standard. The red bonds highlight the newly formed unsaturated bonds generated during the photocatalytic acceptorless dehydrogenation process.

## Data Availability

The original contributions presented in this study are included in the article/Supplementary Material. Further inquiries can be directed to the corresponding author.

## Supplementary Materials

The following supporting information can be downloaded at: https://www.mdpi.com/article/10.3390/ma19122602/s1, Table S1: The atomistic coordinates of PT-COF with AA stacking generated by calculations; Table S2: Influence of solvents in the reaction; Table S3: Control experiments at varying pH; Figure S1: Comparative crystalline stability of PT-COF and Co-PT-COF in various solvents; Figure S2: FE-SEM images of PT-COF and Co-PT-COF; Figure S3: HRTEM/TEM images and EDX mapping of Co-PT-COF; Figure S4: PXRD patterns of PT-COF and Co-PT-COF; Figure S5: XPS spectra of PT-COF and Co-PT-COF; Figure S6: Pore size distribution of PT-COF and Co-PT-COF; Figure S7: TGA curves of PT-COF and Co-PT-COF; Figure S8: Mott–Schottky curves of PT-COF; Figure S9: PL spectra of PT-COF and Co-PT-COF; Figure S10: High-resolution mass spectrometry of 2-methylquinoline; Figure S11: FT-IR spectra of Co-PT-COF before and after photocatalysis; Figure S12: Steady-state emission quenching of PT-COF* with cobaloxime; Figure S13: GC analysis. Figures S14–S29: ^1^H NMR spectroscopy and high-resolution mass spectrometry.
