# Supplementary material for: Proton Reduction Catalyst-Grafted Covalent Organic Frameworks for Visible-Light-Driven Acceptorless Dehydrogenation of Cyclic Amines
Source: Materials (Basel). 2026 Jun 17;19(12):2602. doi: 10.3390/ma19122602 (PMC13304192; doi:10.3390/ma19122602)
Supplement: Supplementary file 1 [file materials-19-02602-s001.zip › materials-4341290-supplementary.pdf]

Proton Reduction Catalyst Grafted Covalent Organic Frameworks for Visible-Light-Driven Acceptorless Dehydrogenation of Cyclic Amines

Bin Wang [1], Xinyu Shi [1], Qianping Wang [1], Xinrui Jiang [1], Wanqi Wang [1], Hui Chen [1] *

1. Instrumentation

2. Methods

3. Supplementary Figures and Table

4. ^{1}H NMR Spectrum

1. Instrumentation

Powder X-ray diffraction (PXRD) data were collected using a D8 diffractometer from Bruker, Germany, at 40 kV and 30 mA with Cu Kα radiation (λ = 1.5406 Å). The scanning range was set from 2° to 40° with a step size of 0.02°. X-ray photoelectron spectroscopy (XPS) data were obtained using a Thermo ESCALAB 250XI spectrometer from Thermo Fisher Scientific, USA. Fourier transform infrared (FT-IR) spectra was recorded using an IRPrestige-21 spectrometer from Shimadzu, Japan, in the range of 500-4000 cm^{-1}, with samples mixed with KBr powder and pressed into pellets for testing. The Brunauer Emmett Teller (BET) specific surface area and porous structure of the materials were measured using a JW-BK200C instrument from JWGB, China, through N_2 physical adsorption, with samples degassed at 120 ℃ for 6 hours prior to testing. The surface morphology of the samples was analyzed using a Gemini SEM500 field emission scanning electron microscope (FE-SEM) from Carl Zeiss, Germany. The lattice fringe spacing of the samples was observed using a Talos F200X high-resolution transmission electron microscope (HR-TEM) from Thermo Fisher, USA. Ultraviolet-visible (UV-Vis) absorption spectra were obtained through diffuse reflectance spectroscopy (DRS) measurements in the range of 200-800 nm using a Lambda850$^+$ instrument from PerkinElmer, USA. Photoluminescence (PL) spectra were analyzed using an F-320 fluorescence spectrophotometer from Tianjin Gangdong, China. Thermal stability data were obtained in the range of 298 K to 1073 K using a TGA/DSC 3+thermogravimetric analyzer from Mettler Toledo, Switzerland, with a heating rate of 10 ℃/min.

2.Methods

2.1 Electrochemical measurement

All photoelectrochemical measurements, including transient photocurrent response, Mott-Schottky plots, and electrochemical impedance spectroscopy (EIS), were carried out on a CHI 660E electrochemical workstation with a conventional three-electrode setup, using 0.5 M aqueous sodium sulfate (Na_2SO_4) solution as the supporting electrolyte. A conductive glass substrate coated with the as-prepared catalyst slurry was used as the working electrode, a platinum foil was employed as the counter electrode, and a saturated Ag/AgCl electrode served as the reference electrode.

2.2 CV measurement

Three electrode systems comprising glassy carbon (3 mm dia.) was used asworking electrode and Ag/AgCl (0.1 M Tetrabutylammonium in acetonitrile) as reference electrode and platinum wire was used as counter electrode, respectively. CVs were recorded in acetonitrile containing 0.1 M Tetrabutylammonium hexafluorophosphate as electrolytic medium. Solutions were purged with argon for 30 minutes before scans and also jacketed during the measurements.

2.3 EPR measurement

Electron paramagnetic resonance radical-trapping spectra were recorded at room temperature on a Bruker Elexsys E500 X-band spectrometer with 34W blue led irradiation source. Key parameters: microwave frequency 9.85 GHz, power 10 mW, modulation amplitude 1 G. 5,5-Dimethyl-1-pyrroline-N-oxide was used as the spin trap. The test solution contained tetrahydroquinoline (0.1 M), Co-PT-COF (8 mg) and DMPO (0.5 M) in deionized water. The solution was loaded into a quartz capillary, irradiated for 120 s and tested. Control experiments were performed under identical conditions without Co-PT-COF, light irradiation, or tetrahydroquinoline, respectively.

3. Supplementary Figures and Table

Table S1. The atomistic coordinates of PT-COF with AA stacking generated by calculations.

**PT-COF**<br>**AA Stacking**<br>**Space group symmetry: *P*1**<br>**a = 53.272 Å, b = 25.047 Å, c = 3.838 Å**<br>***α = 90.00°, β = 89.94°, γ = 90.48°***							
Atom	x	y	z	Atom	x	y	z
C1	53.999	-227.674	0.526	C31	60.349	-217.274	1.825
C2	52.646	-227.594	0.104	C32	60.474	-214.917	1.823
C3	51.892	-228.777	-0.100	C33	62.450	-216.203	1.741
C4	52.032	-226.362	-0.138	C34	63.919	-216.254	1.642
C5	58.129	-218.390	1.868	C35	66.030	-215.078	1.418
C6	54.882	-226.487	0.760	C36	64.643	-215.058	1.542
C7	56.282	-226.639	0.761	H1	52.640	-225.410	0.016
C8	57.125	-225.542	0.968	H2	57.651	-217.361	1.980
C9	56.585	-224.270	1.183	H3	56.743	-227.667	0.593
C10	55.191	-224.111	1.203	H4	58.256	-225.683	0.961
C11	54.354	-225.208	0.999	H5	54.737	-223.081	1.386
C12	57.327	-219.528	1.796	H6	53.222	-225.074	1.026
C13	57.907	-220.789	1.658	H7	56.192	-219.430	1.850
C14	59.304	-220.909	1.603	H8	59.784	-221.938	1.498
C15	60.110	-219.766	1.677	H9	61.244	-219.865	1.633
C16	59.525	-218.496	1.804	H10	55.889	-221.808	1.583
C17	57.020	-221.947	1.527	H11	55.648	-203.573	1.055
C18	53.837	-204.723	0.133	H12	52.729	-207.197	1.168
C19	52.486	-204.638	0.133	H13	53.975	-209.347	1.561
C20	54.556	-203.536	0.731	H14	57.791	-207.248	0.981
C21	53.867	-207.197	1.110	H15	56.536	-205.085	0.572
C22	54.553	-208.391	1.334	H16	58.566	-208.871	1.533
C23	55.947	-208.421	1.282	N1	57.466	-223.151	1.348
C24	56.617	-209.671	1.471	N2	56.617	-209.671	1.471
C25	57.907	-209.793	1.471	N3	61.707	-217.343	1.767
C26	58.544	-211.103	1.622	N4	61.826	-214.997	1.766
C27	57.780	-212.273	1.728	N5	59.742	-216.058	1.852
C28	58.410	-213.517	1.813	N6	68.836	-215.217	1.071
C29	59.809	-213.604	1.792	N7	76.510	-224.268	-2.478
C30	59.938	-211.185	1.611	N8	85.635	-216.857	-1.742

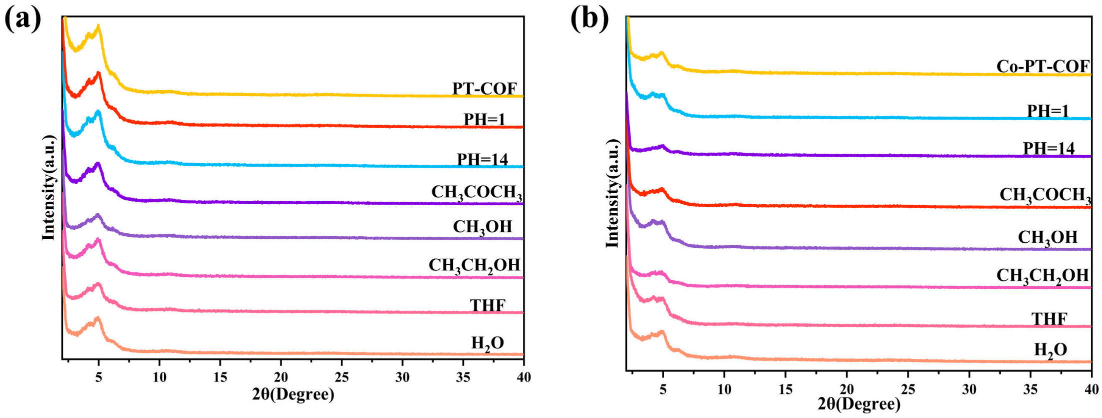

Figure S1. Comparative crystalline stability of (a) PT-COF and (b) Co-PT-COF in various solvents

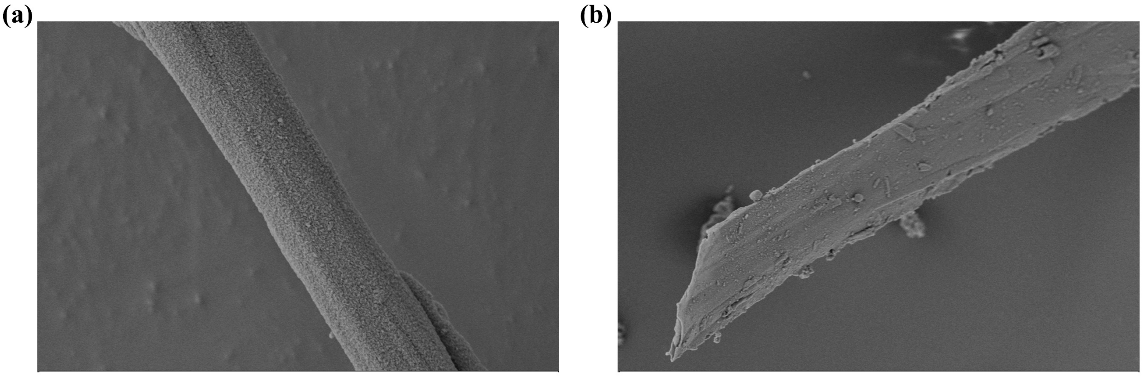

Figure S2. FE-SEM images of (a) PT-COF, (b) Co-PT-COF.

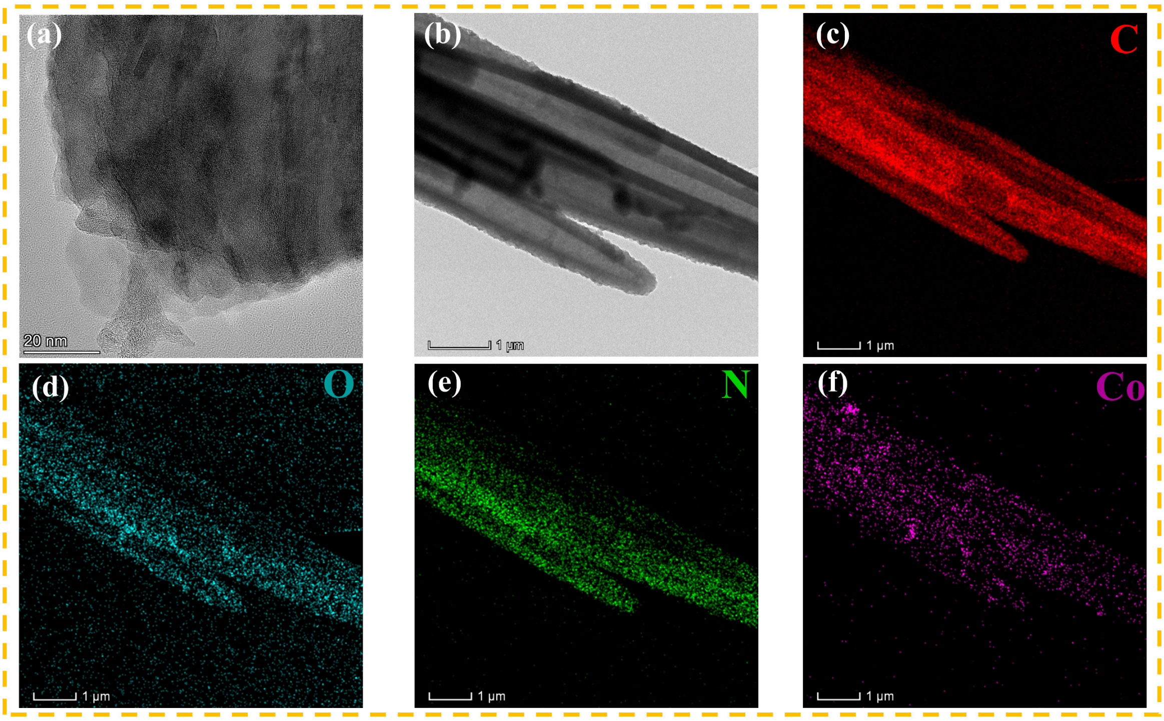

Figure S3. (a) The HRTEM image of Co-PT-COF. (b) The TEM image of Co-PT-COF. (c) ~ (f) The EDX mapping of Co-PT-COF (Co: purple, C: red, O: cyan, N: Green).

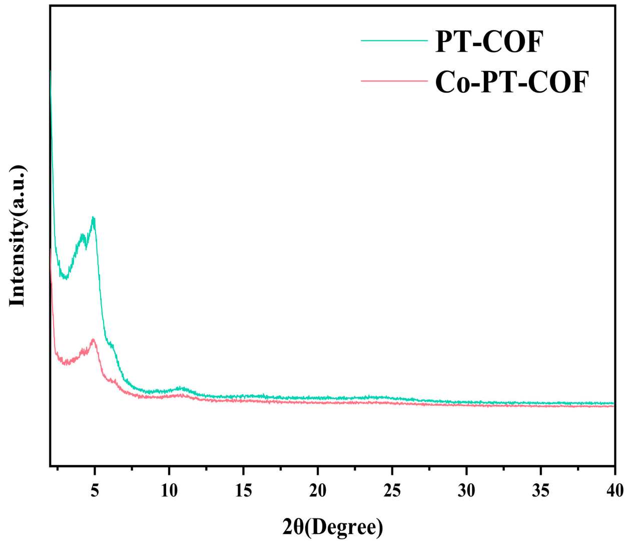

Figure S4. PXRD pattern of experimental PT-COF (green) and Co-PT-COF (pink).

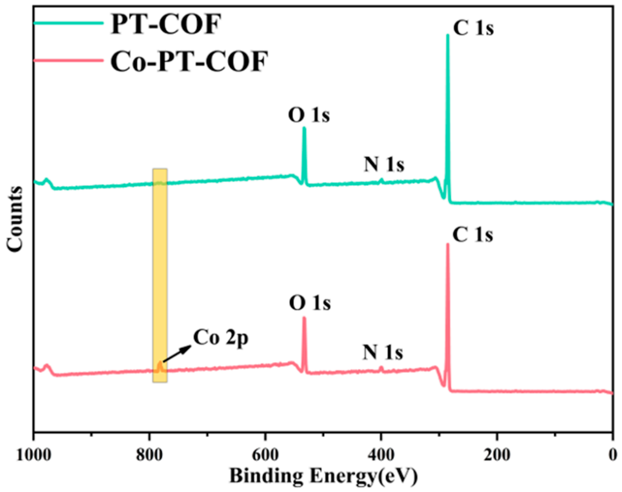

Figure S5. XPS spectra of PT-COF and Co-PT-COF.

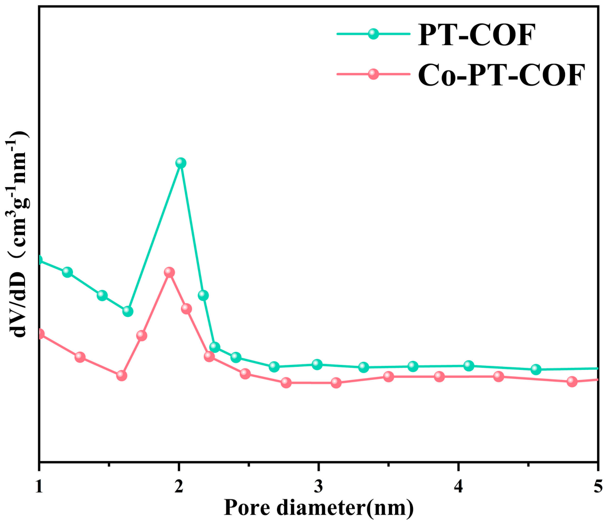

Figure S6. The pore size distribution of PT-COF and Co-PT-COF.

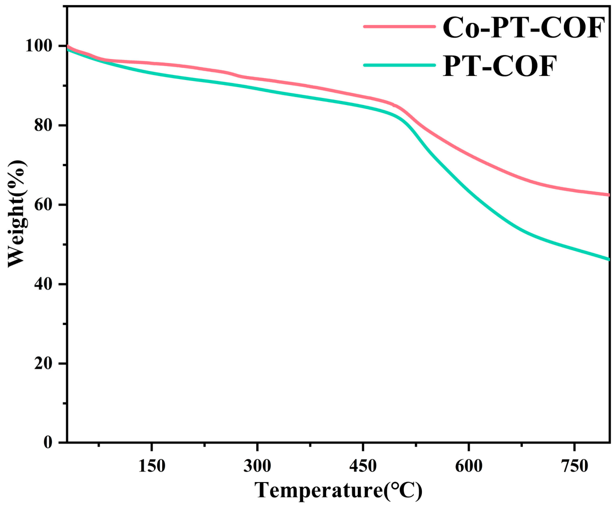

Figure S7. TGA of PT-COF and Co-PT-COF.

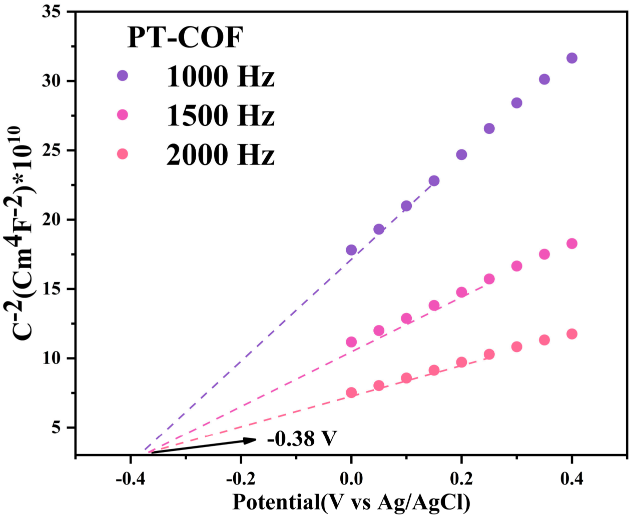

Figure S8. Mott-Schottky curves of PT-COF.

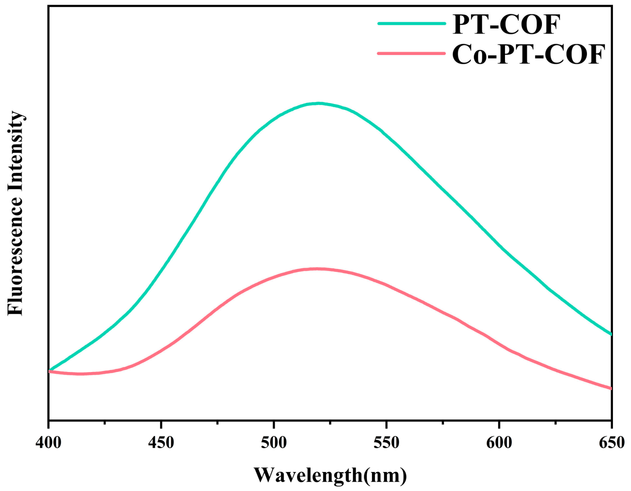

Figure S9. PL spectra of PT-COF and Co-PT-COF.

Table S2. Influence of solvents in the reaction

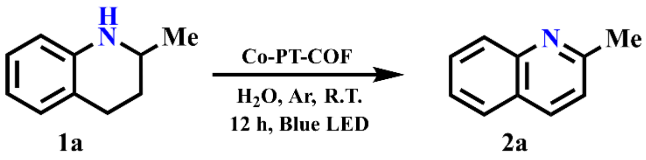

Entry	Variation from the standard conditions	GC yield[b]
1	Methanol instead of water	~ 86%
2	Ethanol instead of water	~ 92%
3	Acetone instead of water	N. R.
4	Acetonitrile instead of water	N. R.
5	Toluene instead of water	N. R.
6	Tetrahydrofuran instead of water	N. R.
7	1,4-Dioxane instead of water	N. R.
8	N,N-Dimethylformamide instead of water	N. R.
9	Dimethyl sulfoxide instead of water	N. R.
10	N-Methylpyrrolidone instead of water	N. R.

Reaction conditions: **1a** (0.25 mmol), Co-PT-COF (10 mg), water (8 mL), Ar atmosphere, visible-light irradiation from blue LED at room temperature for 12 h. [b] GC yield using toluene as an internal standard. N.R. = No Reaction.

Table S3. Control experiments at varying pH

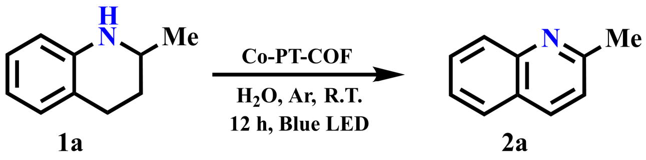

Entry	Variation from the standard conditions	GC yield[b]
1	standard conditions[a]	~ 95%
2	pH = 4	~ 53%
3	pH = 7	~ 92%
4	pH = 9	~ 86%

Reaction conditions: **1a** (0.25 mmol), Co-PT-COF (10 mg), water (8 mL), Ar atmosphere, visible-light irradiation from blue LED at room temperature for 12 h. [b] GC yield using toluene as an internal standard. N.R. = No Reaction.

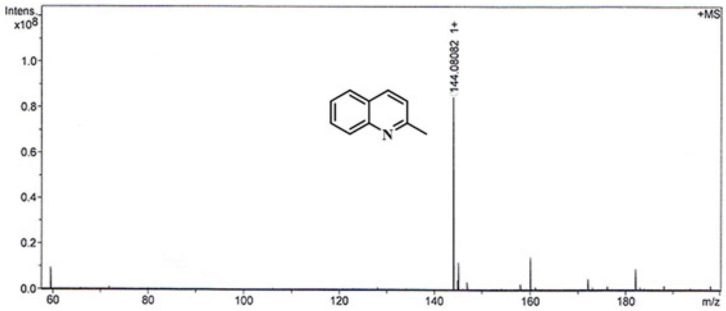

Figure S10. High-resolution mass spectrometry of 2-methylquinoline

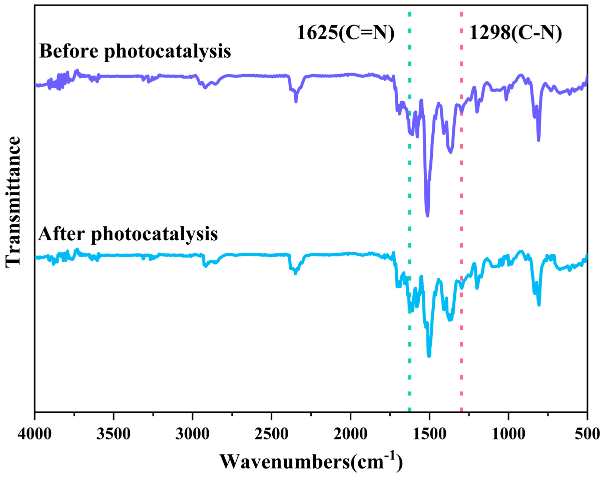

Figure S11. FT-IR spectra of Co-PT-COF before and after photocatalysis.

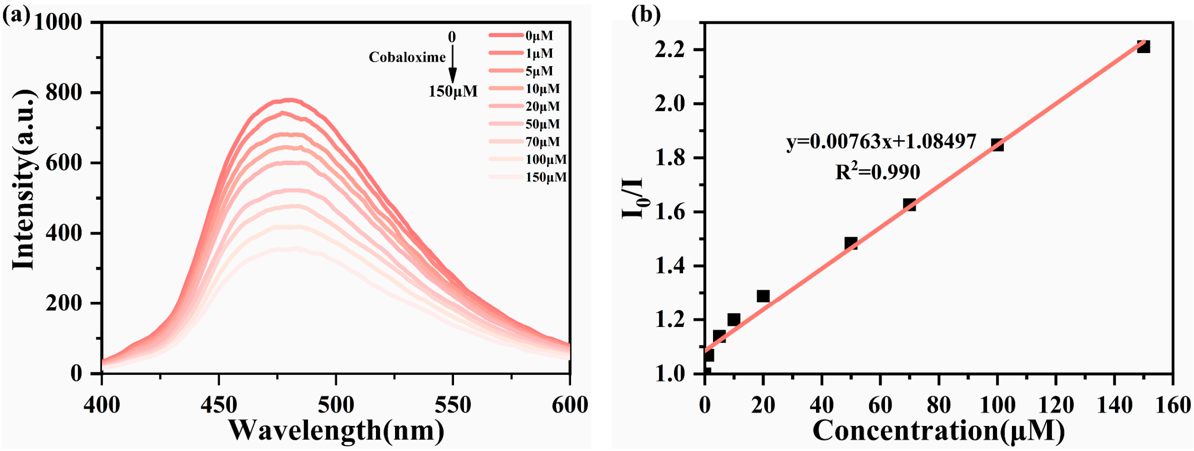

Figure S12. (a) Steady-state emission quenching of PT-COF*with cobaloxime. (b)Stern-Volmer analysis of the results.

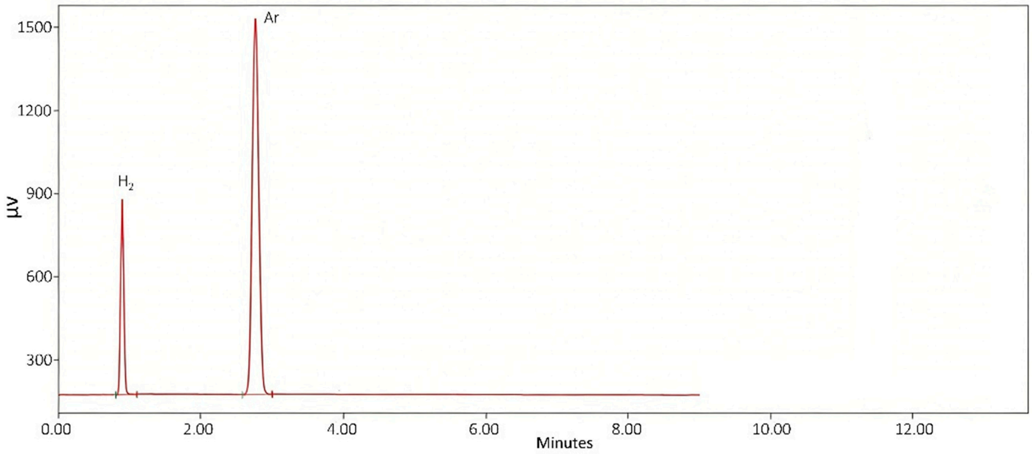

Figure S13. GC analysis.

4. ^{1}H NMR Spectrum

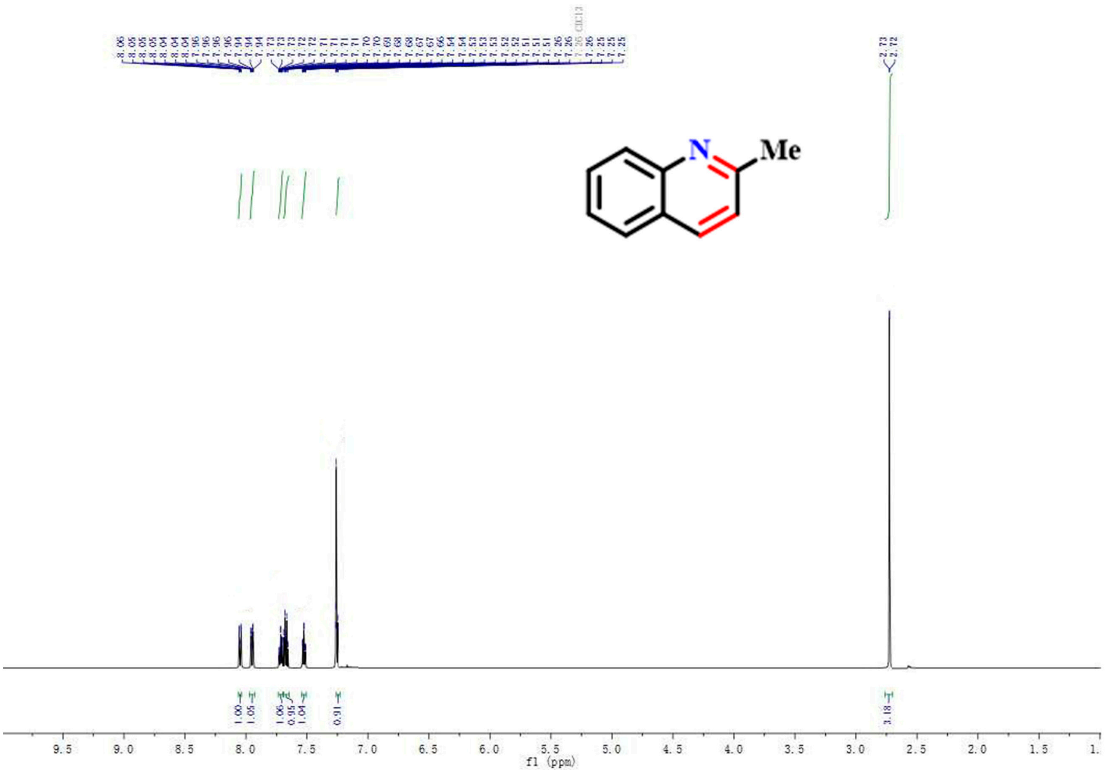

Figure S14. ^{1}H NMR spectrum of 2-methylquinoline.

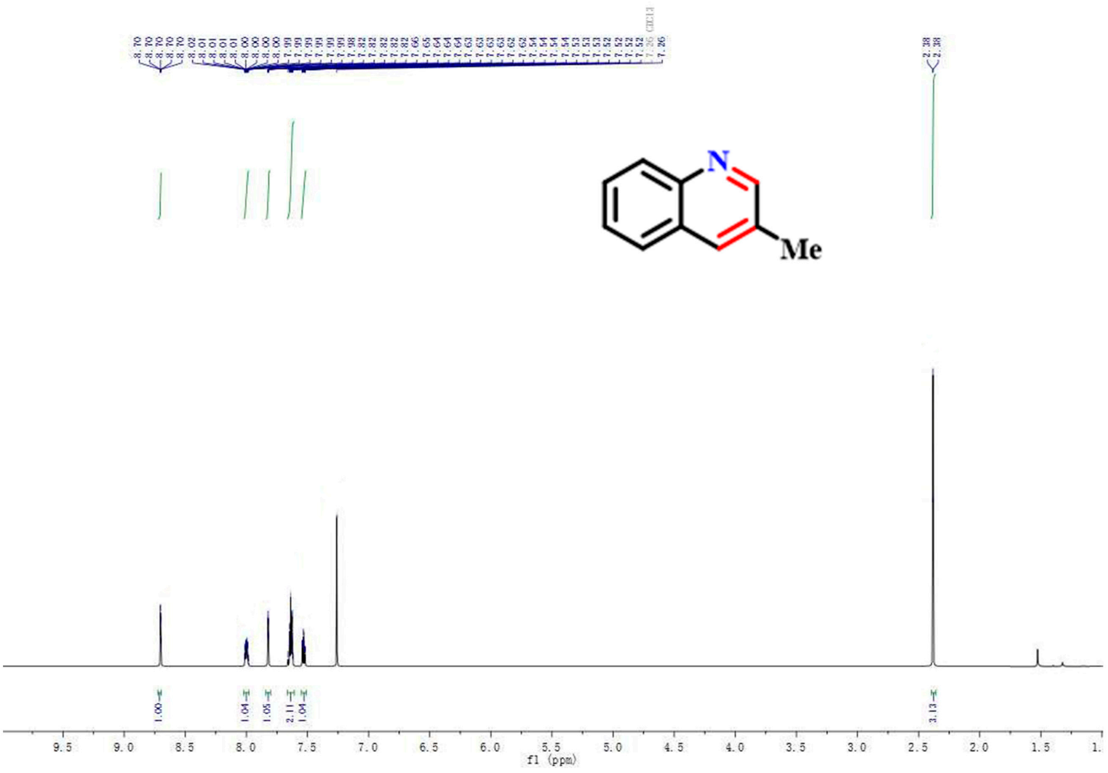

Figure S15. ^{1}H NMR spectrum of 3-methylquinoline.

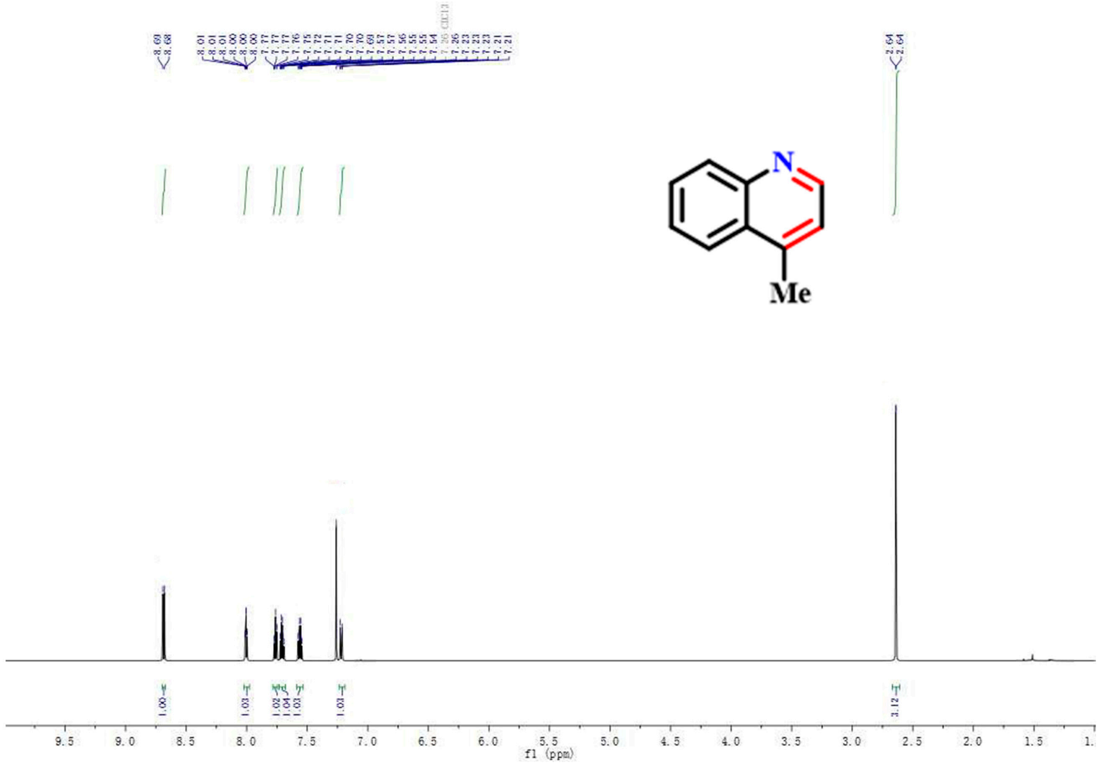

Figure S16. ^{1}H NMR spectrum of 4-methylquinoline.

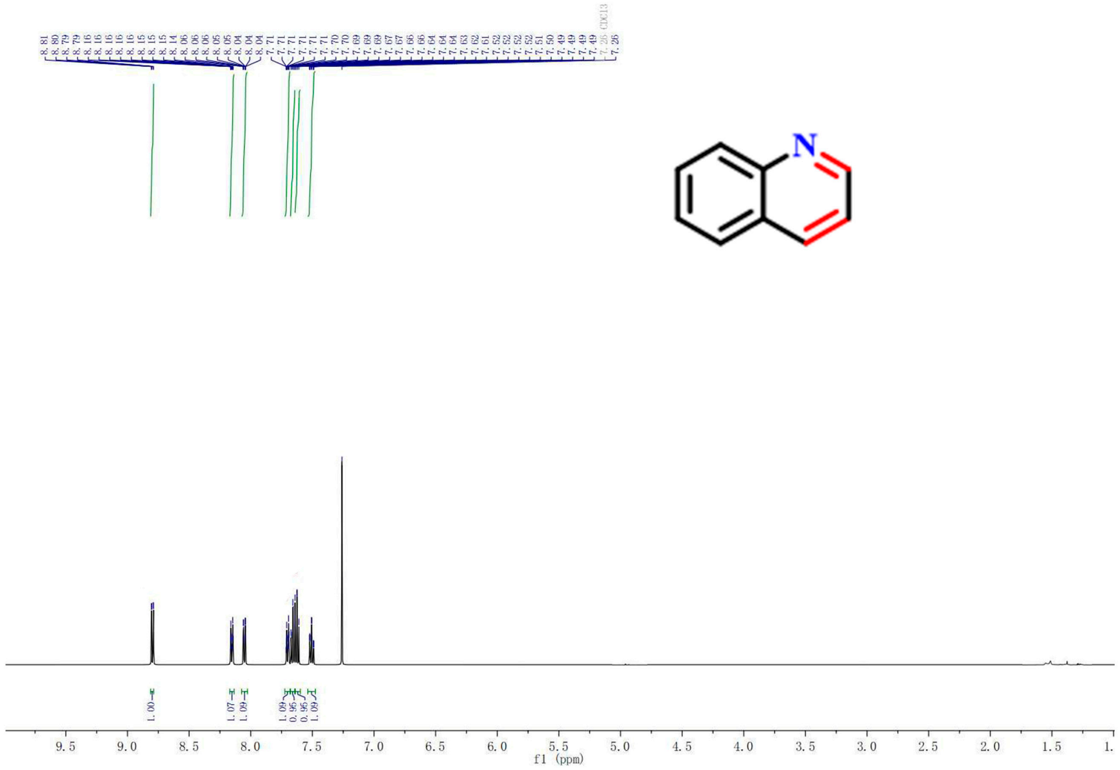

Figure S17. ^{1}H NMR spectrum of quinoline.

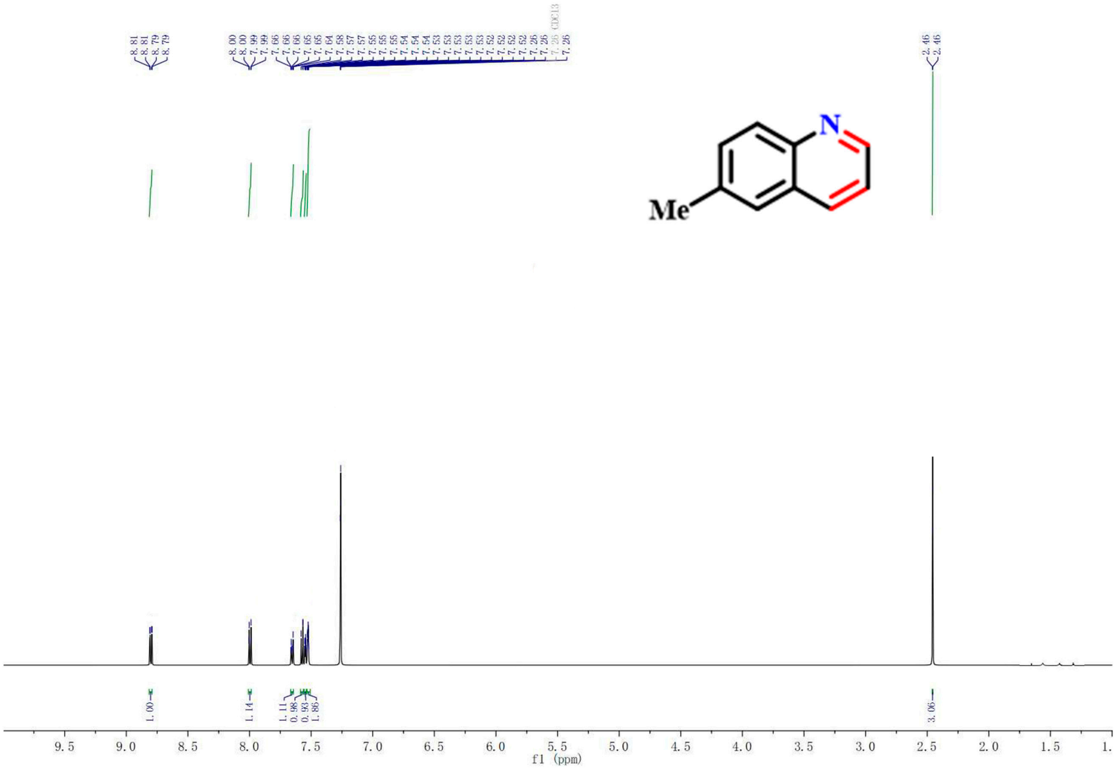

Figure S18. ^{1}H NMR spectrum of 6-methylquinoline.

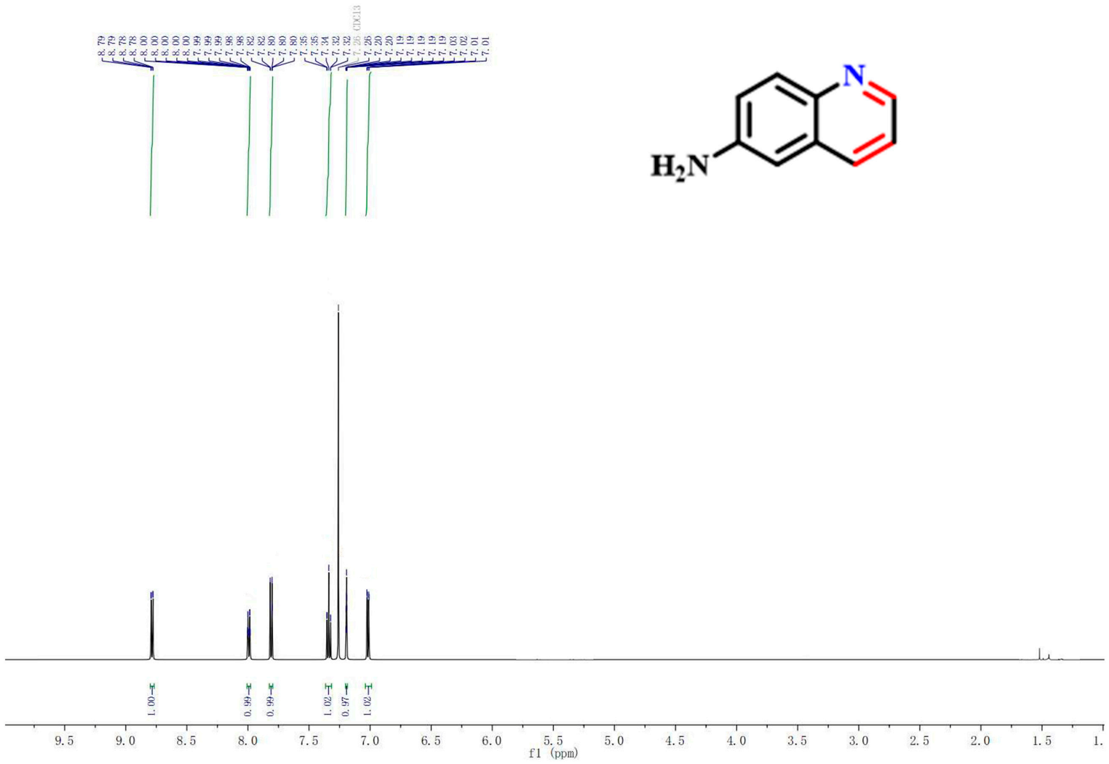

Figure S19. ^{1}H NMR spectrum of 6-aminoquinoline.

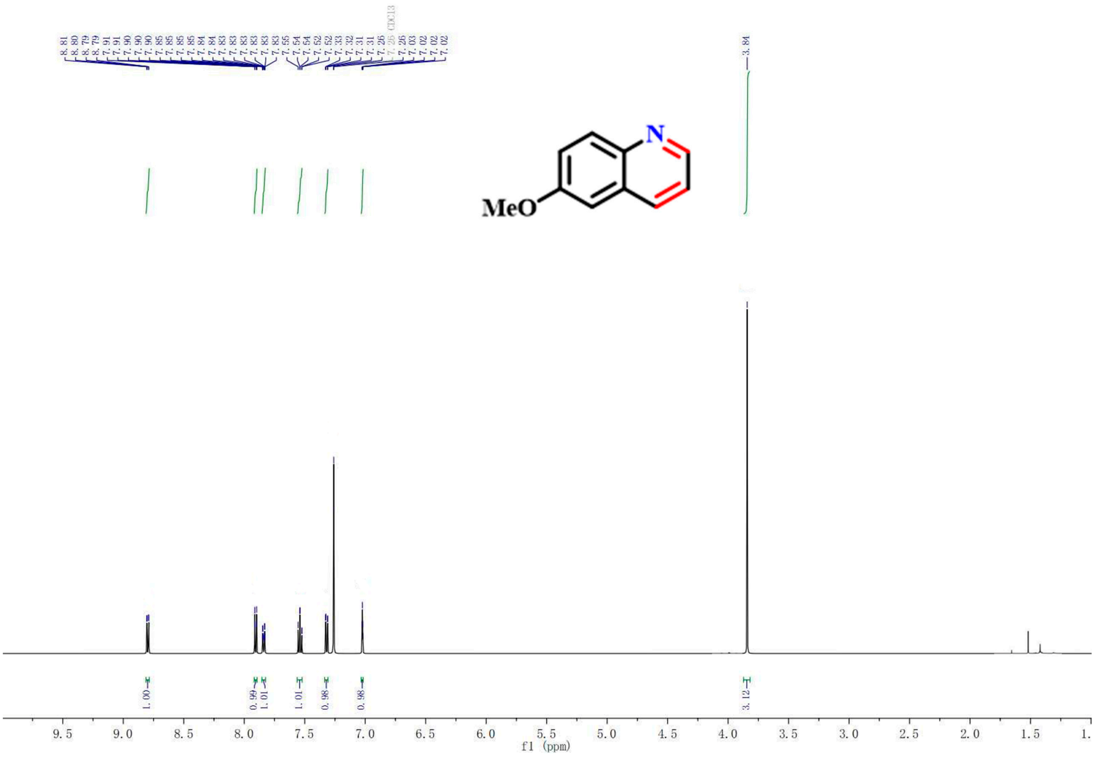

Figure S20. ^{1}H NMR spectrum of 6-methoxyquinoline.

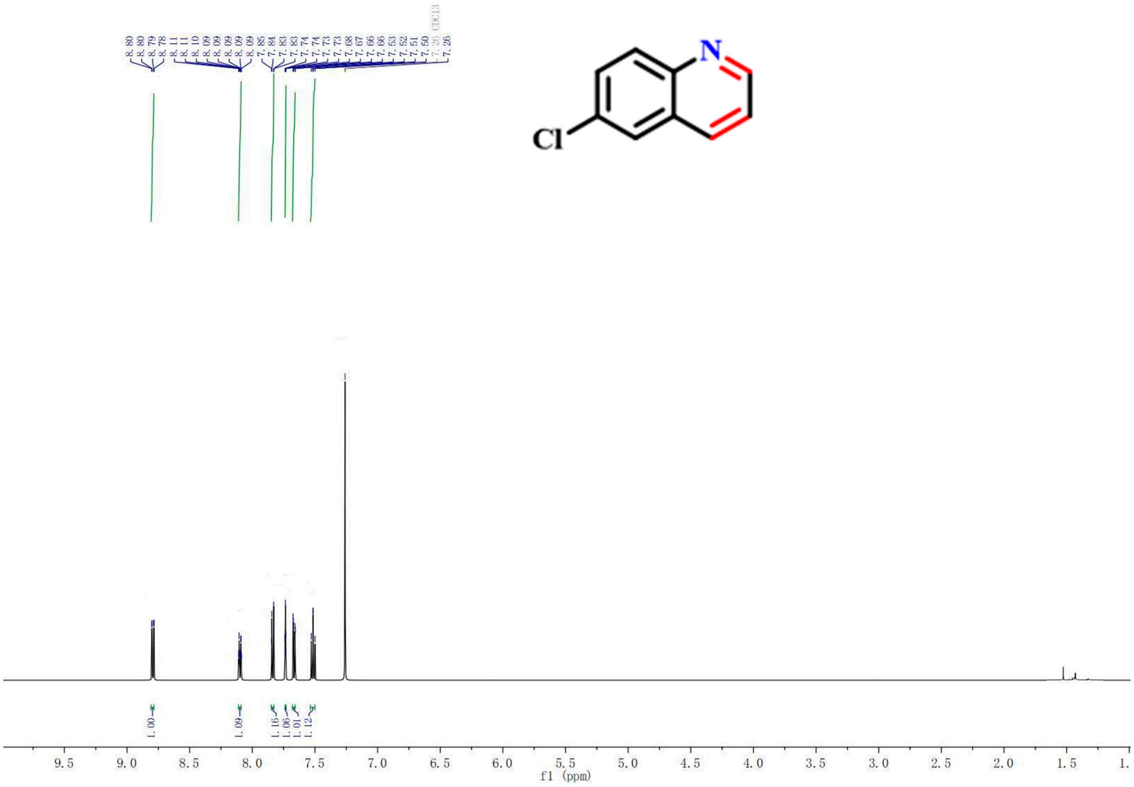

Figure S21. ^{1}H NMR spectrum of 6-chloroquinoline.

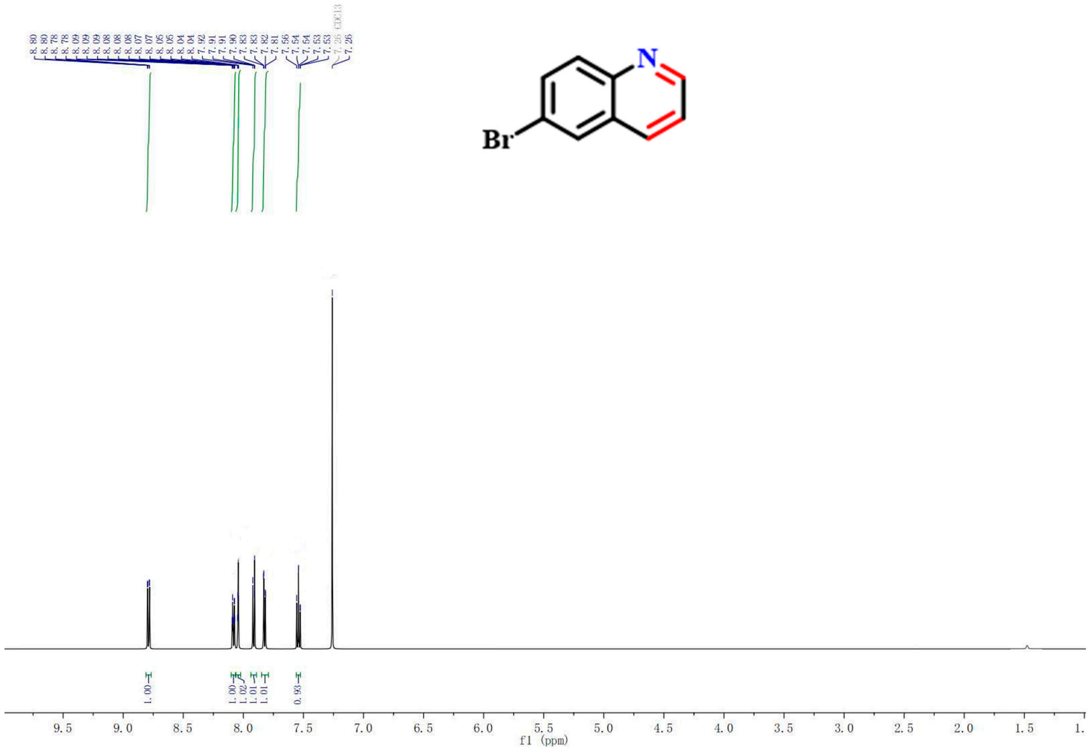

Figure S22. ^{1}H NMR spectrum of 6-bromoquinoline.

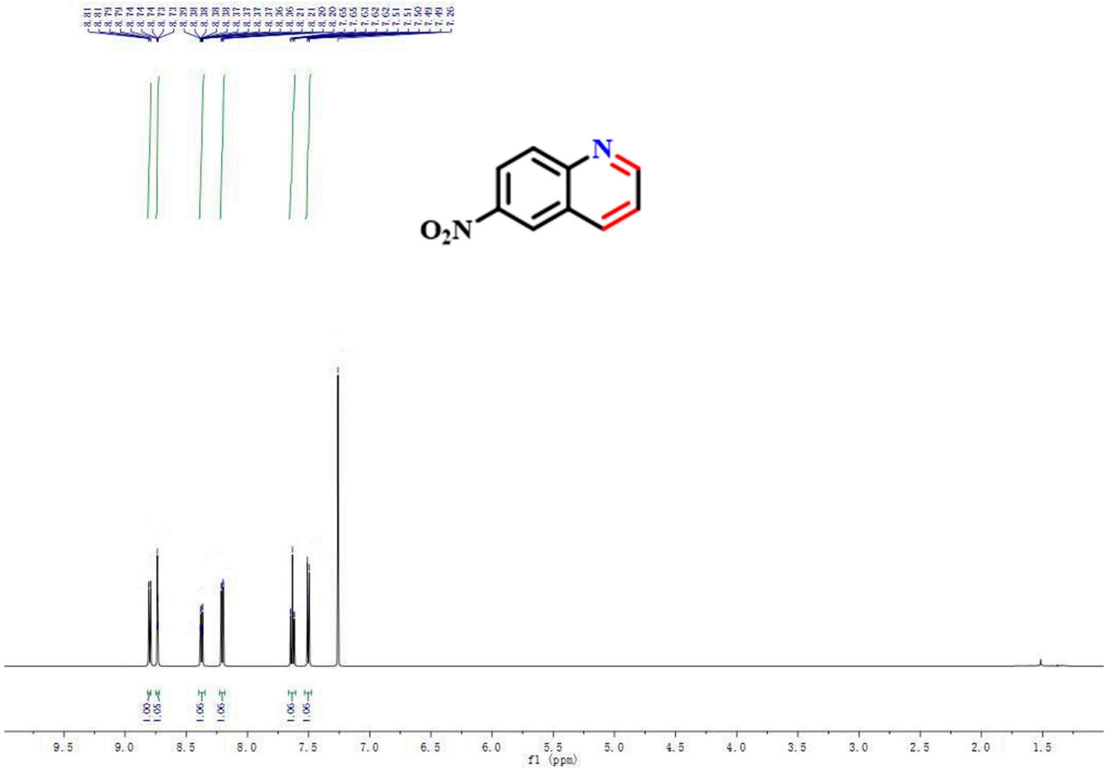

Figure S23. ^{1}H NMR spectrum of 6-nitroquinoline.

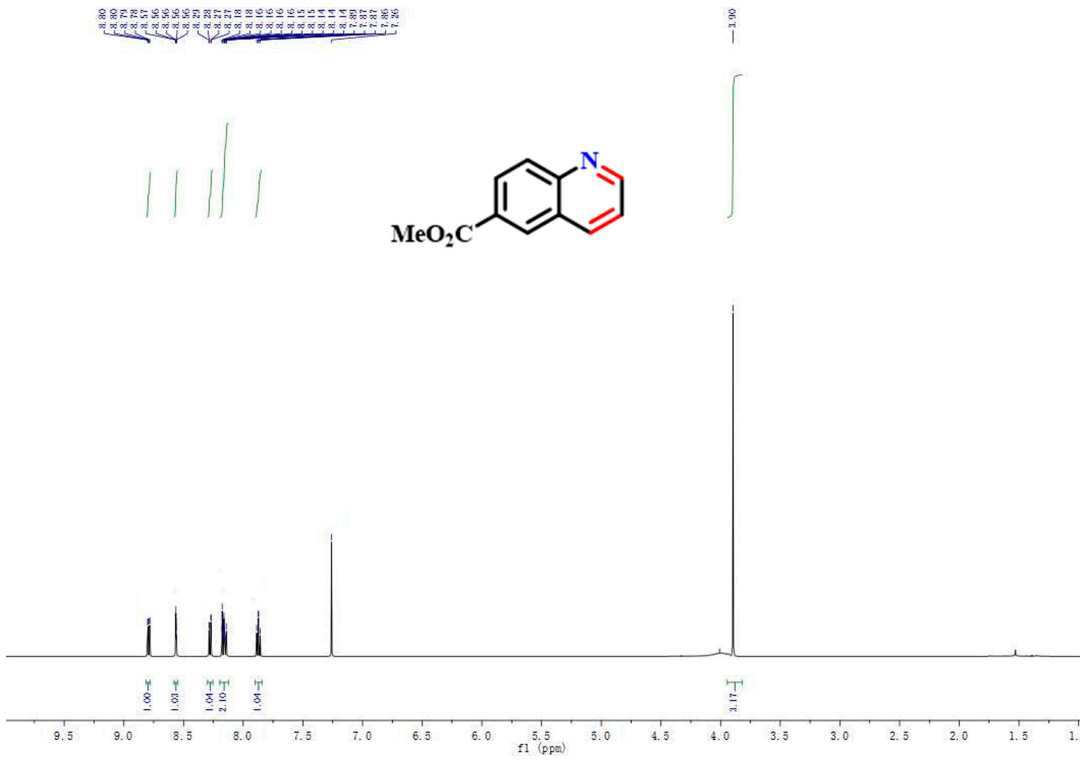

Figure S24. ^{1}H NMR spectrum of methyl quinoline-6-carboxylate.

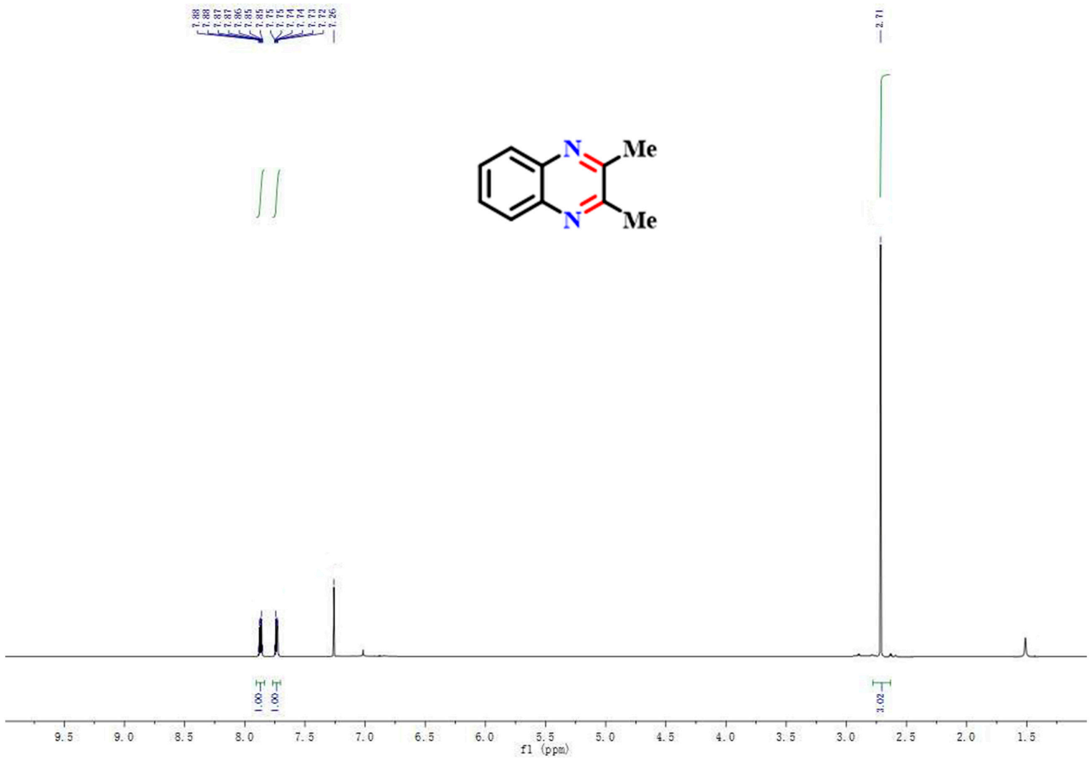

Figure S25. ^{1}H NMR spectrum of 2,3-dimethylquinoxaline.

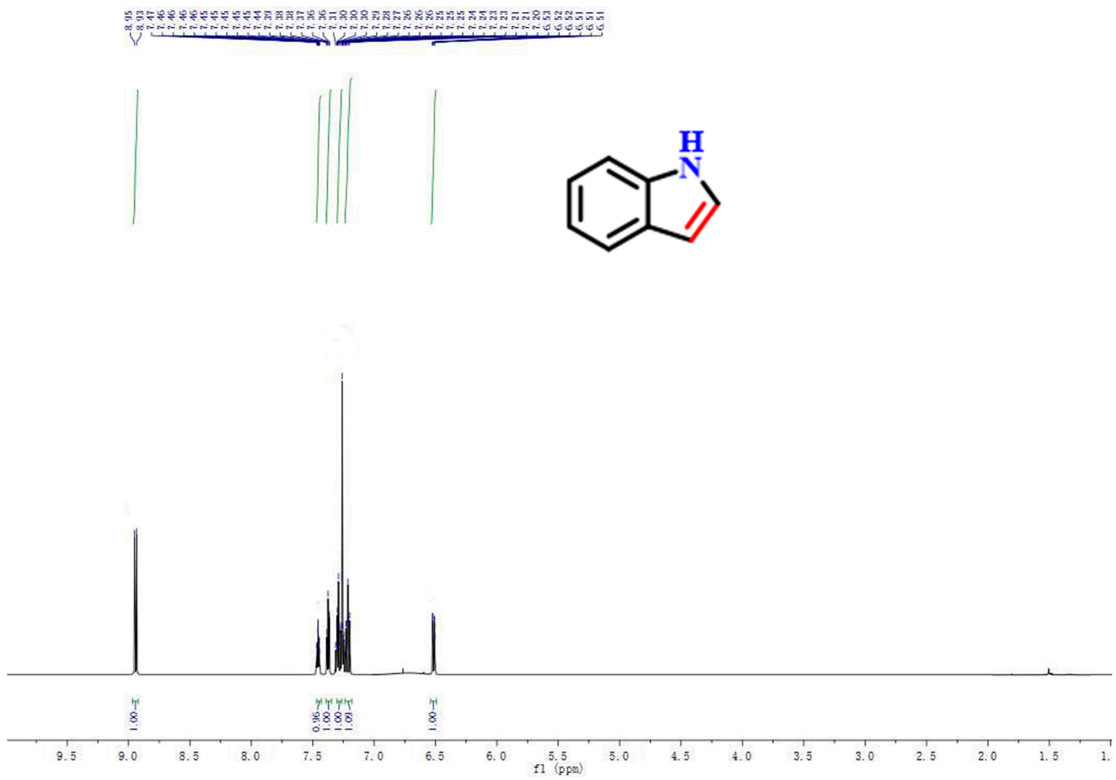

Figure S26. ^{1}H NMR spectrum of indole.

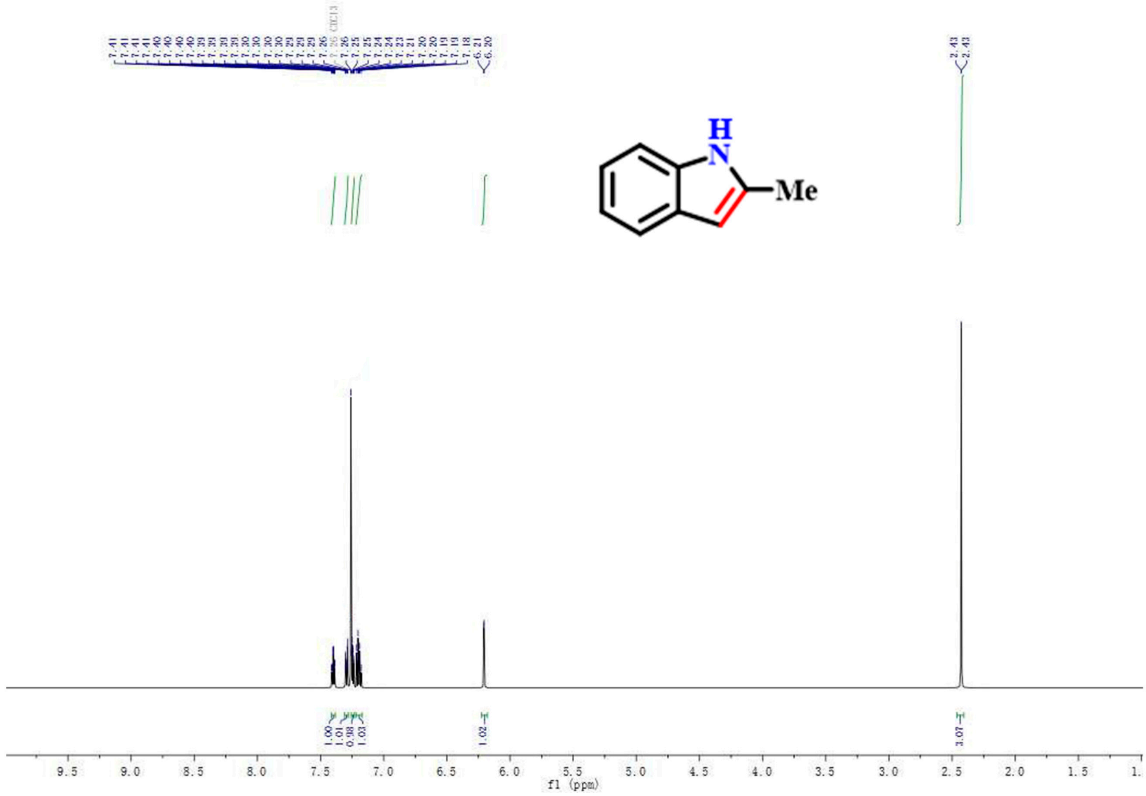

Figure S27. ^{1}H NMR spectrum of 2-methylindole.

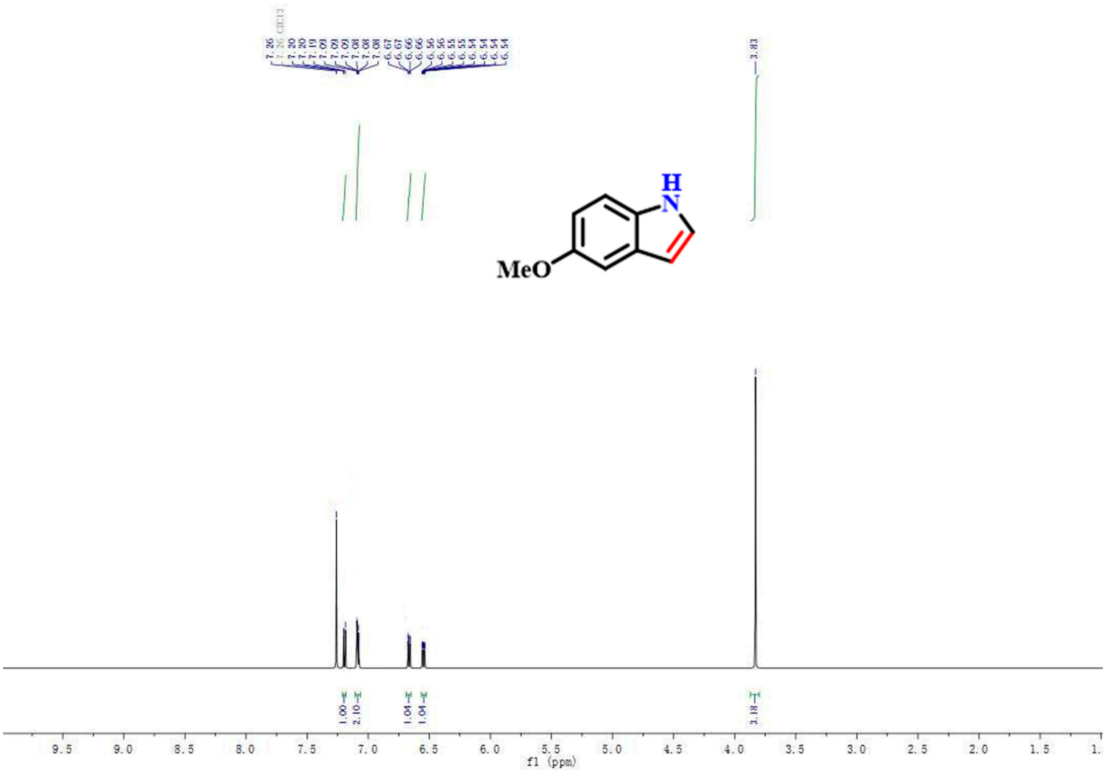

Figure S28. ^{1}H NMR spectrum of 5-methoxyindole.

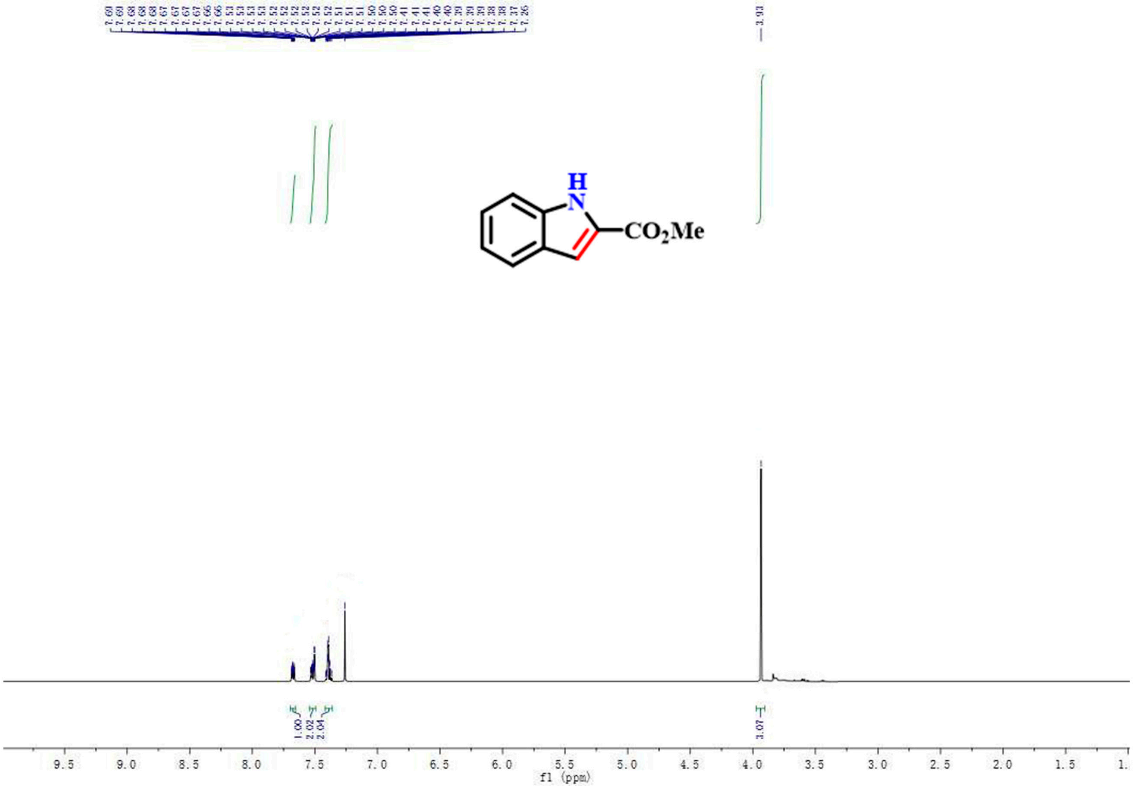

Figure S29. ^{1}H NMR spectrum of methyl 1H-indole-2-carboxylate.
